# Supplementary material for: SPIN reveals genome-wide landscape of nuclear compartmentalization
Source: Genome Biol. 2021 Jan 14;22:36. doi: 10.1186/s13059-020-02253-3 (PMC7809771; doi:10.1186/s13059-020-02253-3)
Supplement: Supplementary file 1 — Additional file 1 Supplementary Methods and Supplementary Results. [file 13059_2020_2253_MOESM1_ESM.pdf]

# Supplementary Methods

## Multi-fraction Repli-seq data generation

Exponentially growing K562 cells (ATCC CCL-243) cultured in IMDM-10% FBS were pulse-labeled with 200 $\mu$M BrdU for 60 minutes, then fixed in 70% ethanol and stained with propidium iodide as previously described for sorting by DNA content [1]. FACS sorting windows were defined as previously published [2]. Briefly, the area between G1 and G2 peaks were divided into 6 windows evenly (S1-S6). Another window (G1) was added immediately on the left of S1 to collect non-replicating cells. At least 120,000 cells were collected from each fraction, then using 120,000 cells each, sequencing libraries were made. From G1 fraction, the total genome library was made as the control. From S1-G2 fraction, libraries that represent nascent DNA were made by performing anti-BrdU immunoprecipitation as described [1]. These libraries were sequenced on HiSeq 2500 with 50SE chemistry aiming to obtain 20-40 million reads each.

## Simulation data generation

To evaluate the performance of SPIN, we generated simulation datasets and directly compared the results with baseline methods such as $K$-means and Gaussian HMM. For simulation, we first generated a graph on chromosome 1 at 25kb resolution where edges in the graph represent the significant Hi-C interactions. We kept the ratio between the local interactions (<1Mb) and long-range interactions (>1Mb) as 3:1, similar to the ratio derived from the real data. Given the graph structure, we used Gibbs sampling to generate a series of simulated states and the corresponding TSA-seq and DamID signals. First, we initialized the graph with random state labels. Then the Gibbs sampling sampled on each node in the graph, one at a time. Gibbs sampling can take advantage of the conditional independence properties of the Markov random field. Thus, when sampling the state $H_i$ of node $i$, we only need to consider its direct neighboring nodes $N(i)$. The state $H_i$ was sampled from distribution: $P(H_i|\{H_j|_{j\in N(i)}\})$. We iteratively updated node state labels until convergence (no changes in state labels). Given the simulated state, TSA-seq and DamID signals on each node were sampled using the distribution learned from the real data and then smoothed. We applied SPIN to estimate the hidden states based on TSA-seq, DamID, and the graph structure as input. As a comparison, we also estimated the states using $K$-means and Gaussian HMM, which only used TSA-seq and DamID signals as input. We evaluated the prediction accuracy of different methods as well as different ways of defining Hi-C interactions and significant interaction $p$-value cutoffs (see Additional file 1: Supplementary Results and Additional file 2: Fig. S18).

## Considering structural variations in K562

Since K562 is a cancer cell line, we also took large structural variants (SVs) into account. For example, if genomic region $s$ and $t$ are adjacent in the cancer genome but not in the normal genome, we would add an edge $(s, t)$ in the HMRF graph structure. We applied our recently developed SV caller Weaver [3] to generate SVs in K562 based on the whole-genome sequencing (WGS) data from the CCLE project [4]. Weaver identified 121 large-scale (allele-specific) SVs in K562 (Additional file 4: Table S2). 64%

of Weaver identified SVs were also reported in [5]. We then integrated these SVs into our HMRF graph construction by adding/deleting edges (see below). Overall, 46 edges due to SVs were added and 29 edges were removed. First, we assigned each breakpoint of SVs to the closest boundary of non-overlapping 25kb genomic bins. We modified the original graph with SV information using the following steps:

1. We call two breakpoints involved in an SV $X$ and $Y$, and the genomic bins upstream/downstream of breakpoint $X$ are referred to as $X_L$ and $X_R$, respectively. Similarly, the genomic bins upstream/downstream of breakpoint $Y$ are referred to as $Y_L$ and $Y_R$.
2. We count the copy number of each bin on two alleles separately (as part of the output from Weaver). We denote the two alleles as A and B, respectively. For example, the copy number of bin $X_L$ on allele A is notated as $C(X_L^{(A)})$ and the copy number on allele B is $C(X_L^{(B)})$.
3. Deletion of edges: for breakpoint $X$, we check the connectivity of the chromosome on each allele based on copy number.

$$Con(X^{(A)}) = \begin{cases} 0, & \text{if } C(X_L^{(A)})) = 0 \\ 0, & \text{if } C(X_R^{(A)})) = 0 \\ 1, & \text{otherwise} \end{cases} \tag{19}$$

   where $Con(X^{(A)})$ stands for the connectivity of allele $A$ at breakpoint $X$. $Con(X^{(A)}) = 0$ indicates that all copies of allele A are completely disconnected at breakpoint $X$. $Con(X^{(A)}) = 1$ indicates that at least one copy of the chromosome is connected at breakpoint $X$. The genome is disconnected at breakpoint $X$ when both alleles are broken. In other words, we will delete edge $(X_L, X_R)$ in the original graph when $Con(X^{(A)}) = 0$ and $Con(X^{(B)}) = 0$. Otherwise, we will keep the original edge because at least one copy of the chromosome is connected near breakpoint $X$. We do the same for breakpoint $Y$.
4. Adding edges. For breakpoints $X$ and $Y$, we calculate the difference in copy numbers across breakpoints. For example, the difference of copy number for breakpoint $X$ is defined as:

$$D(X) = C(X_L^{(A)}) - C(X_R^{(A)}) + C(X_L^{(B)}) - C(X_R^{(B)}) \tag{20}$$

   If $D(X) = 0$, we do not expect SVs at breakpoint $X$. Otherwise, we add edges to the graph to reflect the SV. Typically we only observe the change of copy number on one of the allele. The orientation of edges to add can be determined by $D(X)$ and $D(Y)$.

## Collecting results from other chromatin state annotation methods

We compared the SPIN states with other chromatin state annotations, including Hi-C subcompartments, chromatin state annotations from ChromHMM [6] as well as Segway [7] and Segway-GBR [8].

For Hi-C subcompartments, we used the SNIPER Hi-C subcompartments in K562 [9], although the trend with SPIN state comparison is largely consistent with the original Hi-C subcompartment definitions from GM12878 [10]. We focused on the five primary Hi-C subcompartments, i.e., A1, A2, B1, B2, and B3.

To compare SPIN states with Hi-C subcompartments, we calculated the genome-wide coverage fold change of each SPIN state over each Hi-C subcompartment. For each Hi-C subcompartment, we calculated the total length of regions covered by different SPIN states and normalized it by the expected length, which was calculated by the genome-wide coverage of SPIN states (as shown in Fig. 1c). We then defined the ratio between the observed length and the expected length as the fold change score of the SPIN states. If one SPIN state is uniformly distributed along the genome, its fold change score for each Hi-C subcompartments should be 1. A higher fold change score indicates certain SPIN states are more likely to be found in a given Hi-C subcompartment.

We used the SNIPER Hi-C subcompartment calls in all 9 human cell lines: K562, GM12878, HAP1, HeLa, HMEC, HSPC, HUVEC, IMR90, and T Cells from [9] to estimate the constitutive level of subcompartment annotations. We calculated entropy as a metric for the level of conservation of Hi-C subcompartments across different cell types. Within each 25kb genomic bin, we have the Hi-C subcompartment calling in 9 cell lines, and for each subcompartment $i$, we use $l_i$ to denote the total genomic length across 9 cell lines. The entropy was thus calculated as:

$$E = - \sum_{i \in \{A1,A2,B1,B2,B3\}} \left( \frac{l_i}{L} \right) \log \left( \frac{l_i}{L} \right) \tag{21}$$

where $L$ is the sum of genomic length for all subcompartments in that genomic bin across cell types.

We downloaded ChromHMM and Segway chromatin state annotations in K562 from the ENCODE Project. In K562, there are 25 ChromHMM states and 25 Segway states identified from histone modifications, transcription factor binding, and open chromatin data. We also downloaded the combined chromatin state definition in K562, which is a consensus merged from the results of ChromHMM and Segway [11]. There are seven states in the combined segmentation. We calculated the total length of the overlapping regions between ChromHMM/Segway/Combined and the SPIN states, and then normalized it by the expected length. The expected length was calculated by the genome-wide coverage of the SPIN states. We then calculated the fold enrichment of chromatin states from ChromHMM, Segway, and Combined, respectively, over each SPIN state. For Segway-GBR chromatin domain annotations, we used the original Segway-GBR annotations in [8] for GM12878 and IMR90 cell lines. For fair method comparison with Segway-GBR, instead of using TSA-seq and DamID as input, we ran SPIN using the same input with Segway-GBR in IMR90 and GM12878 cell lines. The input data include 11 histone modifications (H2A.Z, H3K27ac, H3K27me3, H3K36me3, H3K4me1, H3K4me2, H3K4me3, H3K79me2, H3K9ac, H3K9me3, H4K20me1), and DNase-seq. The histone modification and DNase-seq signals were averaged on 25kb bins and reduced dimensions using PCA. The first 5 principal components were used as input for SPIN. For Hi-C, we used H1 and IMR90 Hi-C data from [12] (same as what [8] used). We downloaded 6-fraction Repli-seq in IMR90 and GM12878 from the ENCODE project to evaluate the performance of these two different methods.

### Methods for sequence feature analysis in SPIN states

To identify sequence features associated with SPIN states, we focused on quantifying of *k*-mers, transposable elements (TEs), and TF motifs on SPIN states. For *k*-mers, we counted 6-mer occurrences on

25kb genomic windows using Jellyfish [13]. Then we used *t*-SNE [14] to visualize all possible 6-mers, where each dot represents a 25kb genomic bin with its SPIN states shown in colors (Additional file 2: Fig. S14).

For transposable elements (TEs), we downloaded the annotations of TE families from the UCSC Genome Browser [15]. The 16 TE families that we used are srpRNA, scRNA, snRNA, Alu, MIR, L1, L2, CR1, ERVL, ERV1, Gypsy, ERVK, LTR, DNA, hAT, and PiggyBac. We further grouped the 16 TE families into 5 classes based on their sequence properties (RNA, SINE, LINE, LTR, and DNA). We counted the density of each TE family on different SPIN states and compared to the genome-wide average.

For known TF motifs, we used the 730 human TF motifs from CIS-BP database [16] and scanned on the open chromatin regions of the whole genome for matches of each motif. The open chromatin regions were defined from DNase-seq peaks in K562 from the ENCODE project (accession number: ENCSR921NMD). Peak calling of DNase-seq was done by the ENCODE standard pipeline. We used FIMO with *p*-value cutoff 1E-5 for motif matching. The known TF motifs were further grouped into 12 classes based on the annotation from CIS-BP.

We used MEME [17] for *de novo* motif discovery and motif enrichment analysis. On each SPIN state, we extracted randomly sampled DNA sequences on that SPIN state as the primary sequence set and randomly sampled DNA sequences of the whole genome as the control sequence set. We set the length of motifs to be between 6 and 50 nt. We then used FIMO to find the distribution of discovered motifs. We also used Tomtom to match the identified motifs to known TF motifs. The known motifs used here are the human TF motifs from HOCOMOCO v11.

## Supplementary Results

### Resolution of the genomic bin size in SPIN

Although the SPIN framework can in principle be applied to any resolution (the genomic bin size), here we explain why we selected 25kb as the resolution in this work. The key input data types of SPIN (TSA-seq and DamID) are in resolutions greater than 10kb (Additional file 2: Fig. S16a). For example, the input data of TSA-seq scores are at 20kb resolution. Specifically, we calculated the differences of TSA-seq and DamID signals between a pair of genomic loci as a function of their 1D genomic distance. As shown in Additional file 2: Fig. S16b, the mean and variance of the differences increase as the distance of two genomic loci increases. Among the three Hi-C data resolutions (5kb, 10kb, and 25kb) where the Hi-C contact matrices are readily available, we found that the differences of TSA-seq/DamID quantile scores at 5kb and 10kb resolution are very small ($\Delta$quantile $< 1\%$) and greatly increase at 25kb resolution (>2 times fold change). In addition, higher resolution does not show sharper histone transition at SPIN states boundary (Additional file 2: Fig. S16c). Taken together, we chose 25kb as the resolution for SPIN states in this work.

### Comparison with other genome segmentation methods

We compared SPIN to Segway [7] and ChromHMM [6] results in K562 based on histone modification data (Additional file 2: Fig. S19). We also included combined annotations of Segway and ChromHMM from ENCODE. The fold enrichment of each state is calculated. Overall, we found similar results by comparing to Segway and ChromHMM (Additional file 2: Fig. S19). Most enhancer (Enh-), promoter (Prom), and gene-related states (Gen, Tss) have a stronger presence in the Speckle and Interior_Act states. Quiescent state (Quies) has more involvement in the Lamina state and the repressed polyComb state (Repr) is enriched in the Interior_Repr2 state. For combined annotation of Segway and ChromHMM, we found that TSS, T (transcribed), E (Enhancer), WE (weak enhancer), and PF (promoter flanking) all have a strong presence in the Speckle and Interior_Act states. R (Repressed) state is enriched in the Lamina Interior_Repr and Near_Lm states. These results are consistent with our observations that spatial localization is highly correlated with chromatin states and transcription. Most gene-rich and active chromatin regions are in the Speckle and Interior_Act states. Polycomb/facultative repressed regions are in the Interior_Repr state. Heterochromatin and low transcription activity regions are in the Near_Lm and Lamina states.

We also compared SPIN with Segway-GBR [8], which uses graph-based regularization (GBR) to integrate pairwise chromatin conformation data for genome segmentation (Additional file 2: Fig. S20). In order to compare the two methods, we used the same input data in IMR90 and GM12878 from [8] to run SPIN and set the number of states to be 5. We used six-fraction Repli-seq from ENCODE as a way to evaluate the segmentation performance. We expect that a better segmentation will better stratify Repli-seq data. We found that the states produced by SPIN have a clearer Repli-seq stratification as compared to Segway-GBR. SPIN results can distinguish G1 from S1 and G2 from S4 fraction. In contrast, the Repli-seq patterns are similar between SPC and BRD, QUI and CON in the Segway-GBR results, further

suggesting the advantage of SPIN results.

## Simulation study to evaluate the performance of SPIN

To assess the advantage of using SPIN to identify compartmentalization patterns based on TSA-seq, DamID, and Hi-C, we compared the results from SPIN to the baseline methods $K$-means and Gaussian HMM on the simulated data. We also used this simulation to assess the impact of different ways of defining Hi-C interactions used in the method. We simulated SON TSA-seq, Lamin-B1 TSA-seq, Lamin-B1 DamID, and Nucleolus DamID scores with 25kb window size on chromosome 1 (Additional file 1: Supplementary Methods; Additional file 2: Fig. S18a). In order to generate TSA-seq and DamID scores similar to the real data, we used the 10 SPIN states in K562 as ground truth and used multivariate Gaussian distribution of each state to generate new TSA-seq/DamID scores. The TSA-seq/DamID signals were then smoothed using Hanning window with the length of 21, and used as input for SPIN, $K$-means, and Gaussian HMM. For a fair comparison, we set the number of states to be 10 for $K$-means, Gaussian HMM, and SPIN. In addition, we evaluated different strategies to incorporate Hi-C data as additional edges in the SPIN model. We compared the impact of using significant interactions called from Hi-C O/E data with different $p$-value cutoffs ($10^{-5}$ and $10^{-4}$). We also used Hi-C observed interactions instead of O/E to call significant interactions. Finally, to demonstrate the effect of including Hi-C data, we built a model with only edges between adjacent nodes (i.e., no Hi-C data used) for comparisons. We found that including Hi-C data in SPIN significantly improves the performance for all states (Additional file 2: Fig. S18b-c). In particular, we found that the SPIN without using Hi-C data has much lower accuracy on interior states (Additional file 2: Fig. S18c), mainly because the Interior_Act and Interior_Repr states share similar distribution of TSA-seq and DamID signals which makes it more difficult to distinguish only based on TSA-seq and DamID signals. In addition, using Hi-C O/E data with $p$-value cutoff 10E-5 would have the best performance for SPIN states (0.92 overall accuracy) than the method using O/E with cutoff 10E-4 (0.90 accuracy) and also the method using the observed Hi-C (0.77 accuracy) (Additional file 2: Fig. S18d). We found that using Hi-C observed data as input has little improvement on performance compared with when no Hi-C data is used, further suggesting the importance of incorporating long-range Hi-C O/E signals as input for compartmentalization pattern identification. We also show that SPIN significantly outperforms both $K$-means and Gaussian HMM on all states. These simulation results suggest that by incorporating TSA-seq, DamID, and Hi-C O/E data, SPIN can identify compartmentalization patterns with high accuracy.

## Exploration of incorporating strength of Hi-C interactions as edge weight

We explored the possibility of incorporating strengths of Hi-C interactions into the SPIN framework (Additional file 2: Fig. S21). One way to achieve this is to use Hi-C interaction strength as edge weight. During loopy belief propagation, the message sent between a pair of nodes can be weighted accordingly (weight normalization in Eqn. 14). In this work, edges are treated with equal weight in SPIN. Here we assess whether incorporating strength of Hi-C interactions would make a difference in the SPIN states (Additional file 2: Fig. S21a-c). We explored the possibility to convert Hi-C $\log_2$O/E strength

to edge weights (result example in Additional file 2: Fig. S21b). To assess the overall quality of SPIN states calling, we calculated the enrichment of histone modification and multi-fraction Repli-seq signals on the SPIN states called from the framework with edge weights (Additional file 2: Fig. S21a). We found that the enrichment patterns of histone modification and Repli-seq are similar to the original SPIN states (Fig. 2a and Fig. 3a), while the transition of replication timing (S1 to G2) from interior states to periphery states is smoother in the original SPIN states, suggesting better SPIN states calling from the original results. Overall, we found that incorporating Hi-C strength as edge weights generates very similar SPIN states calling compared with the original SPIN states. For example, over 84% Speckle and 99% Lamina states are overlapped between the original SPIN states and the states called after adding $\log_2$O/E as edge weight (Additional file 2: Fig. S21c).

Next, we looked at the strength of Hi-C O/E interactions across different SPIN states. As shown in Additional file 2: Fig. S21d, the distribution of Hi-C $\log_2$O/E strength is similar across different SPIN states genome-wide. However, when we focused on significant intra-chromosomal Hi-C O/E interactions (edges used in the SPIN framework), we did observe a trend that there are stronger edge weights and more interactions between the same SPIN states. The edge weights and also the number of edges (per 50kb) for the same SPIN states are always the highest, followed by those SPIN states with similar spatial localization context (Additional file 2: Fig. S21e; only Hi-C interactions with distance >100kb are considered). This trend is consistent in the original SPIN states and also the SPIN states after adding edge weight.

These observations suggest that although there is a general trend that genomic bins with higher Hi-C O/E would be more likely to share SPIN states, which is already reflected in SPIN states without adding edges weights, high Hi-C O/E interaction does not necessarily indicate the same state and the interaction could come from SPIN states with similar, but different, spatial localization context.

## TAD-TAD interactions in SPIN states

Recently, [18] showed that long-range TAD-TAD interactions form constitutive TAD cliques and the formation of TAD cliques stabilizes heterochromatin at the nuclear lamina. However, it is unclear how other large TAD cliques are spatially localized and contribute to genome compartmentalization. Here we analyzed TAD-TAD interactions in K562 and compared them to the SPIN states (Additional file 2: Fig. S9). We first used the DI method to call TADs, and we calculated the median of O/E Hi-C interaction between each pair of TADs. For pairs with TADs with median O/E >2, we found that 55.6% are between the same SPIN states, and 31.2% are between similar SPIN states (e.g., between Interior_Act states). The Lamina state has the most self TAD-TAD interaction where over 60% of its interactions are with another TAD in the Lamina state. This is consistent with the observations in [18]. Speckle and Interior_Act3 TADs also tend to self-interact (over 40%). If we separate the TADs into two groups based on their SPIN states, interior (Speckle, Interior_Act, Interior_Repr) and periphery (Near_Lm, Lamina_Like, Lamina), we found that there is only <1% interactions are between interior and periphery TADs. Our results support the hypothesis that TADs form spatially separated cliques. We found that TAD cliques at the Lamina states are the strongest and there are little TAD-TAD interactions between a pair of TADs that

are localized separately at the interior and periphery.

## SPIN states harbor distinct preferences of DNA binding proteins

We explored the DNA binding proteins in different SPIN states based on 381 ChIP-seq datasets in K562 from the ENCODE project. We utilized the conservative peaks provided by ENCODE if available and called peaks using MACS2 [19] if the peaks were not available. For ChIP-seq dataset with replicates, we kept peak regions shared in both replicates. We calculated the ratio of observed and expected length of ChIP-seq peaks in different SPIN states (i.e., the fold enrichment of ChIP-seq peaks in each SPIN state). To find proteins with similar patterns of occupancy in different SPIN states, we performed hierarchical clustering of fold enrichment score for peaks that led to three clusters (Clusters 1-3) (Additional file 2: Fig. S11a). We found that the majority of DNA binding proteins (314; >80%) preferentially bind to the Speckle and Interior_Act states (Cluster 2). Examples include CTCF, SMC3, SP1, and STAT1. 22 of them (including BRCA1, SRSF9, RBM17, and NONO) have ChIP-seq peaks enriched in the Lamina and Near_Lm2 states (Cluster 1). There are also 46 DNA binding proteins (including AGO1, U2AF1, KLF16, and FOXA1) that show preference in binding to Interior_Act2 and Interior_Act3 states (Cluster 3). This clustering analysis demonstrates that even though most of the proteins preferentially bind to transcriptionally active SPIN states, there are many that bind to more target genes in the transcriptionally less active SPIN states (e.g., Interior_Act2 and 3). We also showed that the majority of RBPs have binding sites in the Speckle state (Additional file 2: Fig. S22).

We then asked whether the target genes of the DNA binding proteins in different spatial clusters based on the SPIN states exhibit functional enrichment. For each cluster, we merged all ChIP-seq peaks called by MACS2, and used the merged list of peaks as input for GREAT [20]. As shown in Additional file 2: Fig. S11a and Additional file 5: Table S3, the most enriched GO terms in different clusters vary, suggesting different roles of these DNA binding proteins and their target genes in the entire transcriptional regulatory network.

## SPIN facilitates the identification of sequence features for nuclear compartmentalization

We asked what sequence features are enriched in different SPIN states. We analyzed the distribution of all known human TF motifs (and then grouped by families) on different SPIN states using CIS-BP motifs [16]. We found that some motif families (POU, Forkhead, Hemedomain, SOX, and Rel) are highly enriched in the Lamina and Near_lm2 states ($p$-value <2.2E-16) (Additional file 2: Fig. S11b). Motif families such as ETS, bHLH, Nuclear receptor, and C2H2 have more presence in the Speckle state ($p$-value <2.2E-16). These enriched TF motifs suggest that the sequence motifs and also the corresponding TF families may also play roles in cooperatively modulating global spatial localization.

We next sought to identify TF binding motifs *de novo* in different SPIN states. Here we used DNA sequences of each SPIN state as input for motif discovery using MEME [17] (Additional file 2: Fig. S12). We identified 15 motifs enriched in different SPIN states (Additional file 2: Fig. S12), and matched them to known human motifs (Additional file 2: Fig. S13). Specifically, for the Speckle state, we identified three motifs matched to the known motifs of MAZ, ZN467, VEZF1, WT1, SP1, SP2 and KLF5 with

*p*-value <10E-5 (Additional file 2: Fig. S12). These motifs belong to the C2H2 Zinc Finger families that are enriched in the Speckle state (Additional file 2: Fig. S11b) and have been shown to play roles in transcriptional activation [21].

Earlier studies have shown that transposable elements (TEs) correlate with nuclear localization [22–24]. We assessed the association of TE families in each SPIN state based on repeat families with over 1000 occurrences on the whole genome that were subsequently grouped into five groups (see Additional file 1: Supplementary Methods). For each TE family, we calculated the fold enrichment of repeat counts on each SPIN state compared to the expected (Additional file 2: Fig. S11c). We found that different TE families have different preferred spatial localization. All three small RNAs (srpRNA, scRNA, snRNA) are highly enriched in the Speckle and Interior_Act states (fold enrichment >1.5, *p*-value<2.2E-16), and depleted in other states. For the SINE family, Alu is mostly abundant in the Speckle state (fold enrichment 2.6) followed by the Interior_Act states (fold enrichment >1.5). There is also enrichment of L1 elements in the Lamina and Interior_Repr2 states (*p*-value=3.3E-6), consistent with previous report that L1 elements have higher density on LADs [22]. In addition, ERVK has significant abundance in the Lamina_Like and Interior_Repr1 states (*p*-value<2.2E-16). Taken together, these results further demonstrate the difference in sequence features associated with different SPIN states, suggesting their potential roles in modulating nuclear compartmentalization globally.

## Analysis of three consecutive SPIN states that reflect trajectory

We sought to ask what type of chromatin trajectories that the SPIN states can reveal. We used three consecutive SPIN states to describe the spatial arrangement of the chromatin trajectory in the nucleus (Additional file 2: Fig. S15). For example, a three consecutive Lamina–Near_Lm–Speckle region suggests a trajectory of a chromatin fiber at nuclear lamina on one end and at nuclear speckle on the other end. For each genomic region of a SPIN state, we counted its adjacent SPIN states and merge symmetrical three consecutive patterns. We ignored all genomic fragments with size smaller than 100kb. We found that all Speckle states are flanked by Interior_Act1. Similarly, almost all Lamina states are flanked by Near_Lm2. We found that for genomic regions with the same SPIN state but different three connective patterns, the histone modification and Repli-seq could be different. For example, for Interior_Act states, the histone modification and Repli-Seq signal can be different depending on whether adjacent states include the Interior_Repr or Near_Lm states. The average signal of active histone marks is 1.5 times higher if Interior_Act is not adjacent to the Interior_Repr states. These results strongly suggest that the trajectories of chromatin (or the context of SPIN state) have important implications on the functional roles of the chromatin.

## References


[1] Marchal C, Sasaki T, Vera D, Wilson K, Sima J, Rivera-Mulia JC, et al. Genome-wide analysis of replication timing by next-generation sequencing with E/L Repli-seq. Nature Protocols. 2018;13(5):819.

[2] Hansen RS, Thomas S, Sandstrom R, Canfield TK, Thurman RE, Weaver M, et al. Sequencing newly replicated DNA reveals widespread plasticity in human replication timing. Proceedings of the National Academy of Sciences. 2010;107(1):139–144.

[3] Li Y, Zhou S, Schwartz DC, Ma J. Allele-specific quantification of structural variations in cancer genomes. Cell Systems. 2016;3(1):21–34.

[4] Barretina J, Caponigro G, Stransky N, Venkatesan K, Margolin AA, Kim S, et al. The Cancer Cell Line Encyclopedia enables predictive modelling of anticancer drug sensitivity. Nature. 2012;483(7391):603.

[5] Dixon JR, Xu J, Dileep V, Zhan Y, Song F, Le VT, et al. Integrative detection and analysis of structural variation in cancer genomes. Nature genetics. 2018;50(10):1388.

[6] Ernst J, Kellis M. ChromHMM: automating chromatin-state discovery and characterization. Nature Methods. 2012;9(3):215.

[7] Hoffman MM, Buske OJ, Wang J, Weng Z, Bilmes JA, Noble WS. Unsupervised pattern discovery in human chromatin structure through genomic segmentation. Nature Methods. 2012;9(5):473.

[8] Libbrecht MW, Ay F, Hoffman MM, Gilbert DM, Bilmes JA, Noble WS. Joint annotation of chromatin state and chromatin conformation reveals relationships among domain types and identifies domains of cell-type-specific expression. Genome Research. 2015;25(4):544–557.

[9] Xiong K, Ma J. Revealing Hi-C subcompartments by imputing inter-chromosomal chromatin interactions. Nature Communications. 2019;10.

[10] Rao SS, Huntley MH, Durand NC, Stamenova EK, Bochkov ID, Robinson JT, et al. A 3D map of the human genome at kilobase resolution reveals principles of chromatin looping. Cell. 2014;159(7):1665–1680.

[11] Hoffman MM, Ernst J, Wilder SP, Kundaje A, Harris RS, Libbrecht M, et al. Integrative annotation of chromatin elements from ENCODE data. Nucleic Acids Research. 2012;41(2):827–841.

[12] Dixon JR, Selvaraj S, Yue F, Kim A, Li Y, Shen Y, et al. Topological domains in mammalian genomes identified by analysis of chromatin interactions. Nature. 2012;485(7398):376.

[13] Marçais G, Kingsford C. A fast, lock-free approach for efficient parallel counting of occurrences of k-mers. Bioinformatics. 2011;27(6):764–770.

[14] Maaten Lvd, Hinton G. Visualizing data using t-SNE. Journal of Machine Learning Research. 2008;9(Nov):2579–2605.

[15] Hinrichs AS, Karolchik D, Baertsch R, Barber GP, Bejerano G, Clawson H, et al. The UCSC genome browser database: update 2006. Nucleic Acids Research. 2006;34(suppl_1):D590–D598.

[16] Weirauch MT, Yang A, Albu M, Cote AG, Montenegro-Montero A, Drewe P, et al. Determination

and inference of eukaryotic transcription factor sequence specificity. Cell. 2014;158(6):1431–1443.

[17] Bailey TL, Boden M, Buske FA, Frith M, Grant CE, Clementi L, et al. MEME SUITE: tools for motif discovery and searching. Nucleic Acids Research. 2009;37(suppl_2):W202–W208.

[18] Paulsen J, Ali TML, Nekrasov M, Delbarre E, Baudement MO, Kurscheid S, et al. Long-range interactions between topologically associating domains shape the four-dimensional genome during differentiation. Nature Genetics. 2019;51(5):835.

[19] Zhang Y, Liu T, Meyer CA, Eeckhoute J, Johnson DS, Bernstein BE, et al. Model-based analysis of ChIP-Seq (MACS). Genome Biology. 2008;9(9):R137.

[20] McLean CY, Bristor D, Hiller M, Clarke SL, Schaar BT, Lowe CB, et al. GREAT improves functional interpretation of cis-regulatory regions. Nature Biotechnology. 2010;28(5):495.

[21] Chen C, Zhou Y, Zhou Z, Sun X, Otto KB, Uht RM, et al. Regulation of KLF5 involves the Sp1 transcription factor in human epithelial cells. Gene. 2004;330:133–142.

[22] Guelen L, Pagie L, Brasset E, Meuleman W, Faza MB, Talhout W, et al. Domain organization of human chromosomes revealed by mapping of nuclear lamina interactions. Nature. 2008;453(7197):948–951.

[23] Rhind N, Gilbert DM. DNA replication timing. Cold Spring Harbor Perspectives in Biology. 2013;5(8):a010132.

[24] Yang Y, Gu Q, Zhang Y, Sasaki T, Crivello J, O'Neill RJ, et al. Continuous-trait probabilistic model for comparing multi-species functional genomic data. Cell Systems. 2018;7(2):208–218.

[25] Chen Y, Zhang Y, Wang Y, Zhang L, Brinkman EK, Adam SA, et al. Mapping 3D genome organization relative to nuclear compartments using TSA-Seq as a cytological ruler. J Cell Biol. 2018;217(11):4025–4048.

[26] Xiao R, Chen JY, Liang Z, Luo D, Chen G, Lu ZJ, et al. Pervasive Chromatin-RNA Binding Protein Interactions Enable RNA-Based Regulation of Transcription. Cell. 2019;178(1):107–121.
