# Supplementary material for: SPIN reveals genome-wide landscape of nuclear compartmentalization
Source: Genome Biol. 2021 Jan 14;22:36. doi: 10.1186/s13059-020-02253-3 (PMC7809771; doi:10.1186/s13059-020-02253-3)
Supplement: Supplementary file 2 — Additional file 2 Figure S1–S22. [file 13059_2020_2253_MOESM2_ESM.pdf]

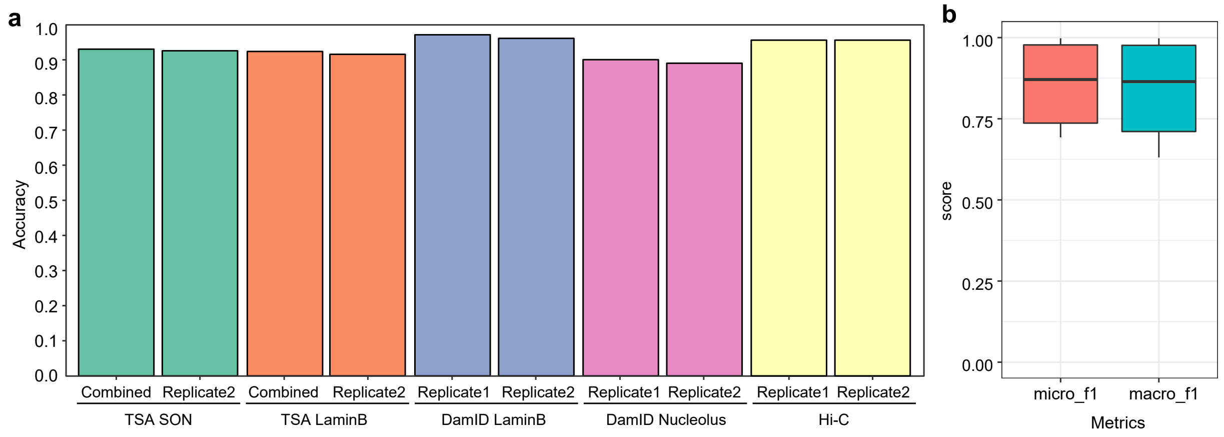

Fig. S1: Robustness analysis of SPIN results. **a.** Consistency of SPIN states based on different “replicate datasets” as input. To test the robustness of SPIN states, we replaced one of the input TSA-seq, DamID or Hi-C with its “replicate” and compared the results with the SPIN states used in this work. In this work, we used dataset for SON TSA-seq and Lamin-B1 TSA-seq generated in [25]. The more recent datasets for SON TSA-seq and Lamin-B1 TSA-seq in K562 generated later with a slightly different protocol were treated as “Replicate 2”. We also have the “Combined” datasets based on the two replicates for both SON TSA-seq and Lamin-B1 TSA-seq. For DamID, we have two technical replicates for both Lamin-B1 DamID and nucleolus DamID (referred to as “Replicate 1” and “Replicate 2”, respectively). In this work, we used the combined DamID data for both Lamin-B1 DamID and nucleolus DamID as input. The two replicates of Hi-C data in K562 were downloaded from 4DN Data Portal (accession numbers 4DNFI9G9FRJJ and 4DNFI4DGNY7J). Here X-axis shows which input data is replaced for SON TSA-seq, Lamin-B1 TSA-seq, Lamin-B1 DamID, nucleolus DamID, and Hi-C. Y-axis shows the accuracy of the SPIN states compared with the original SPIN states used in the paper. **b.** Distribution of micro/macro-F1 scores of randomly initialized model.

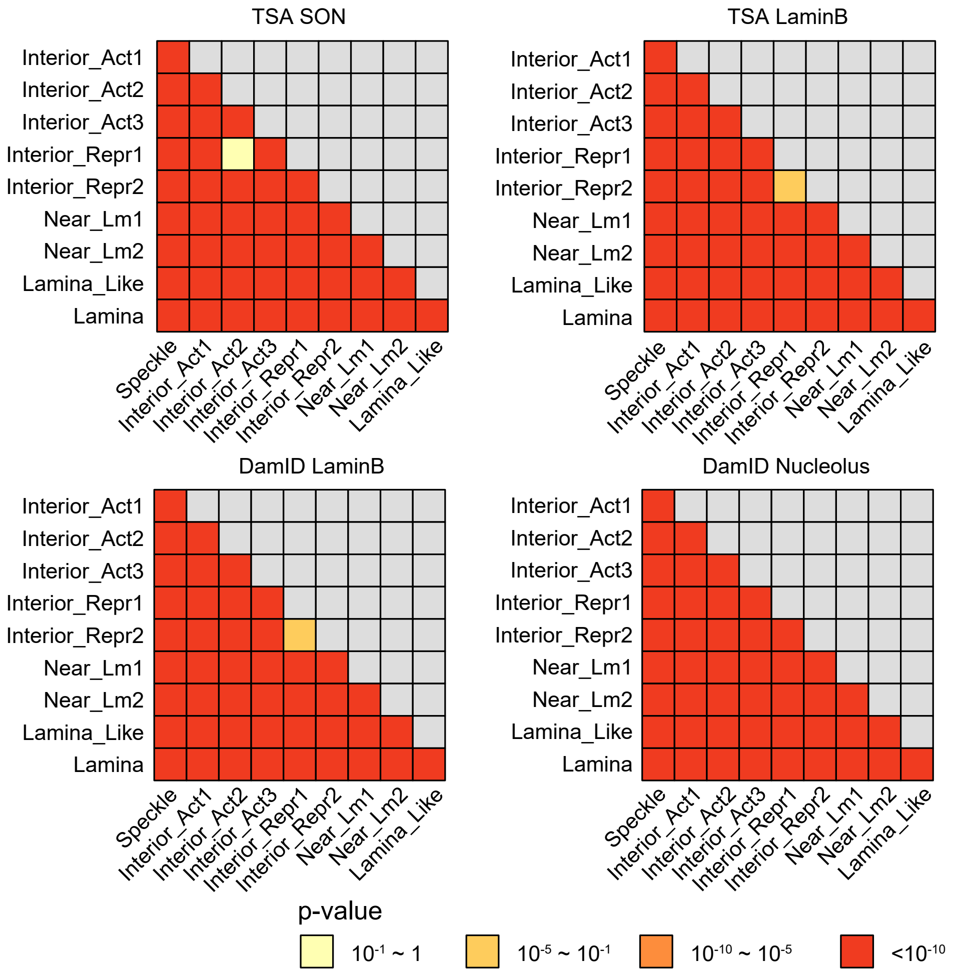

Fig. S2: Wilcoxon tests on TSA-seq and DamID signals for different pairs of SPIN states shown in Fig. 1c. Bonferroni corrected *p*-values are grouped into 4 intervals and are shown in different colors. The majority pairs of SPIN states exhibit significant differences (*p*-value$< 10^{-10}$) in terms of TSA-seq and DamID signal distributions.

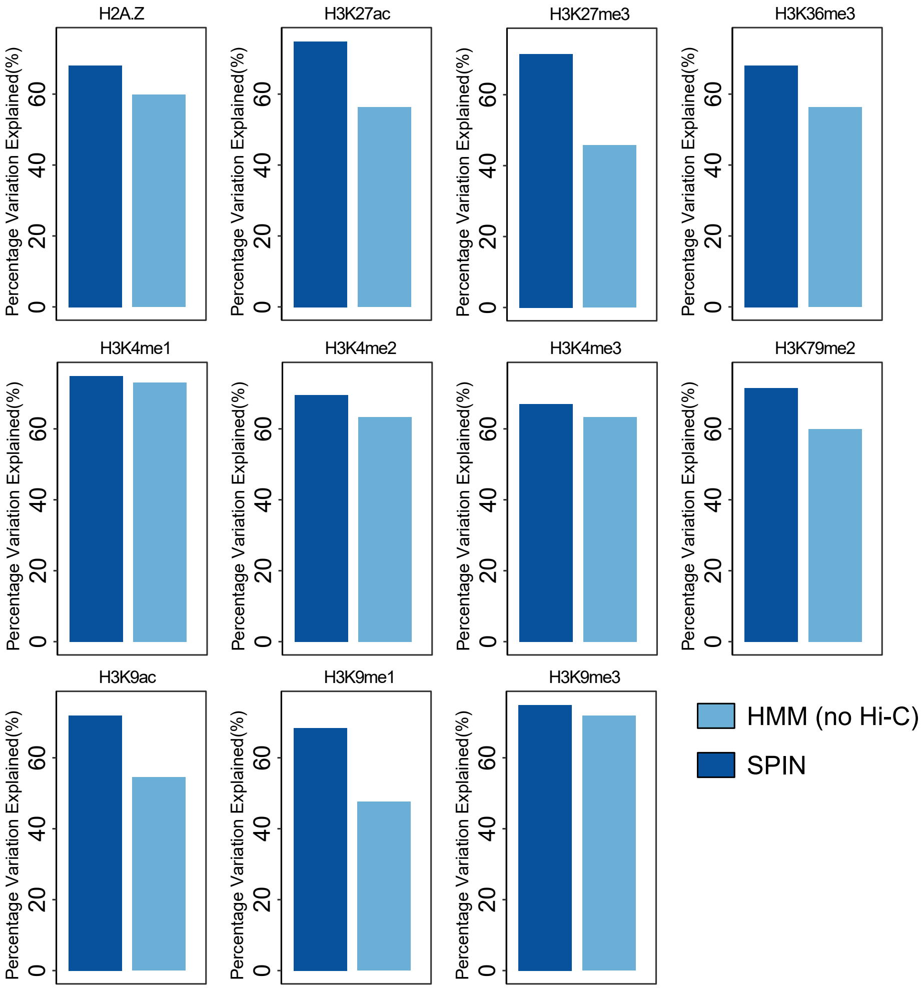

Fig. S3: Percentage of variance for histone modification explained by the SPIN states as compared to the baseline HMM model. The HMM uses the same input (TSA-seq and DamID data) with the SPIN model except for Hi-C, and outputs the same number of states.

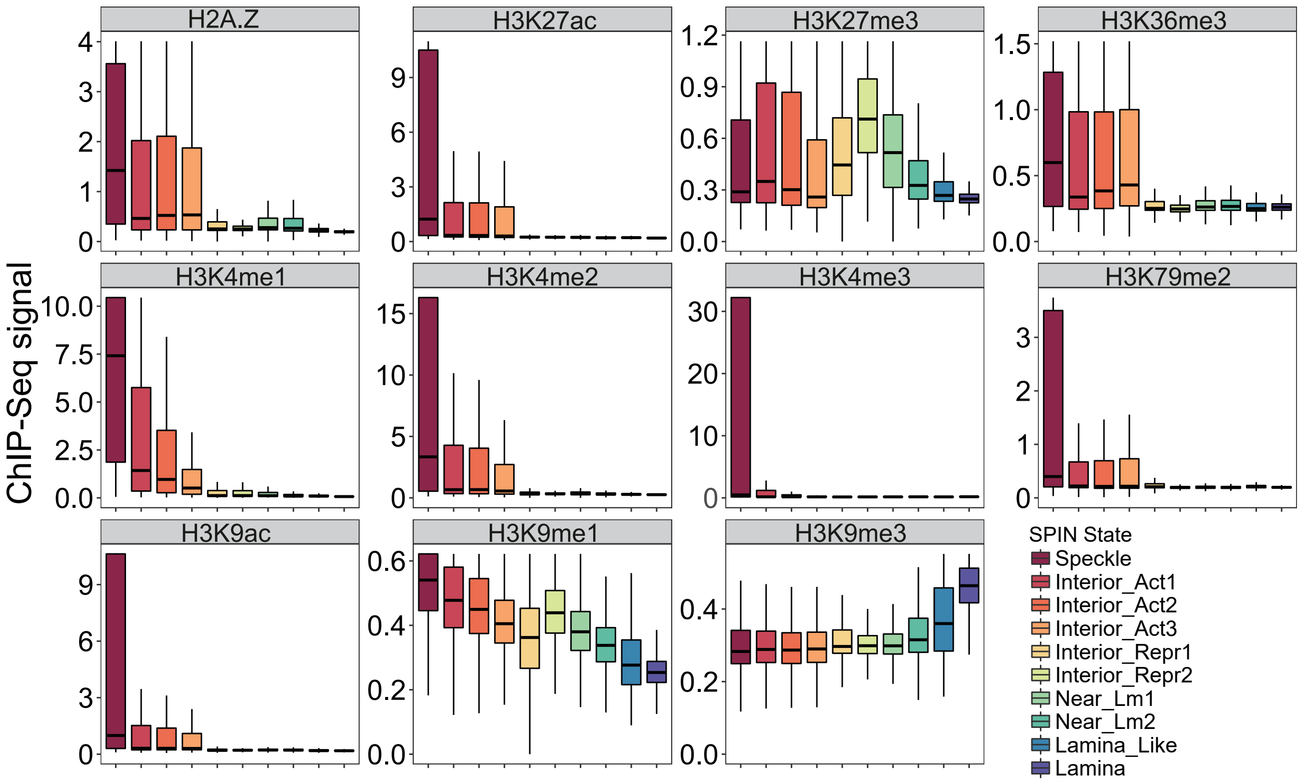

Fig. S4: Distribution of histone modification signals on different SPIN states. 11 histone modification ChIP-seq data in K562 are shown: H2A.Z, H3K4me1, H3K9me1, H3K9me3, H3K27me3, H3K9ac, H3K36me3, H3K4me2, H3K27ac, and H3K79me2. The X-axis shows the histone modification ChIP-seq signal p-value calculated by MACS2.

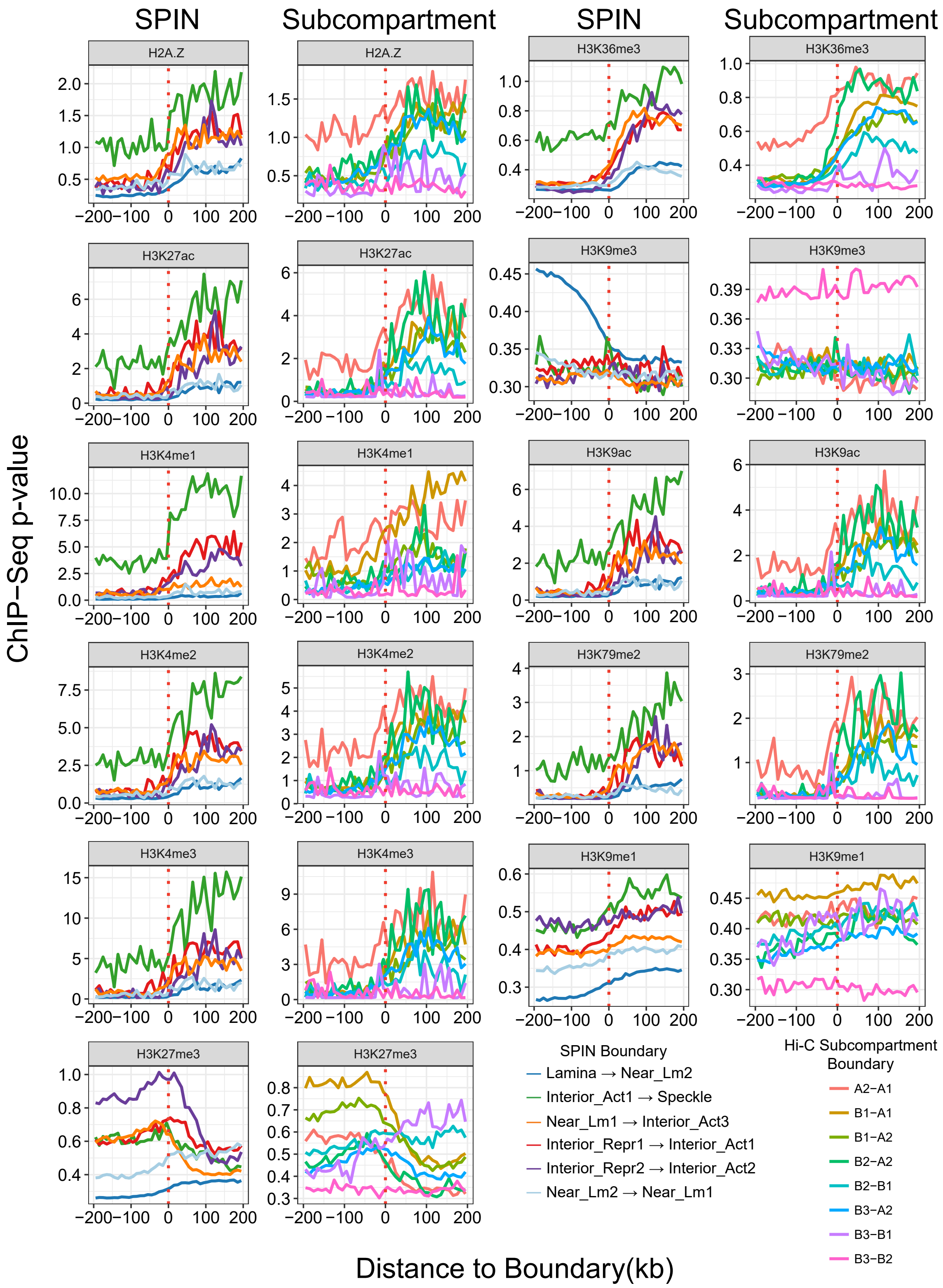

Fig. S5: Histone modification signals at the SPIN states boundaries compared with the Hi-C subcompartment boundaries in K562 (shown side by side). Histone marks here include: H2A.Z, H3K27ac, H3K27me3, H3K36me3, H3K4me1, H3K4me2, H3K4me3, H3K79me2, H3K9ac, H3K9me1, and H3K9me3. Hi-C subcompartment annotations in K562 are from SNIPER [9]. Histone mark signals at most common SPIN and Hi-C subcompartment boundaries are shown. Y-axis shows ChIP-seq p-values for different histone marks. X-axis shows distance to state boundaries.

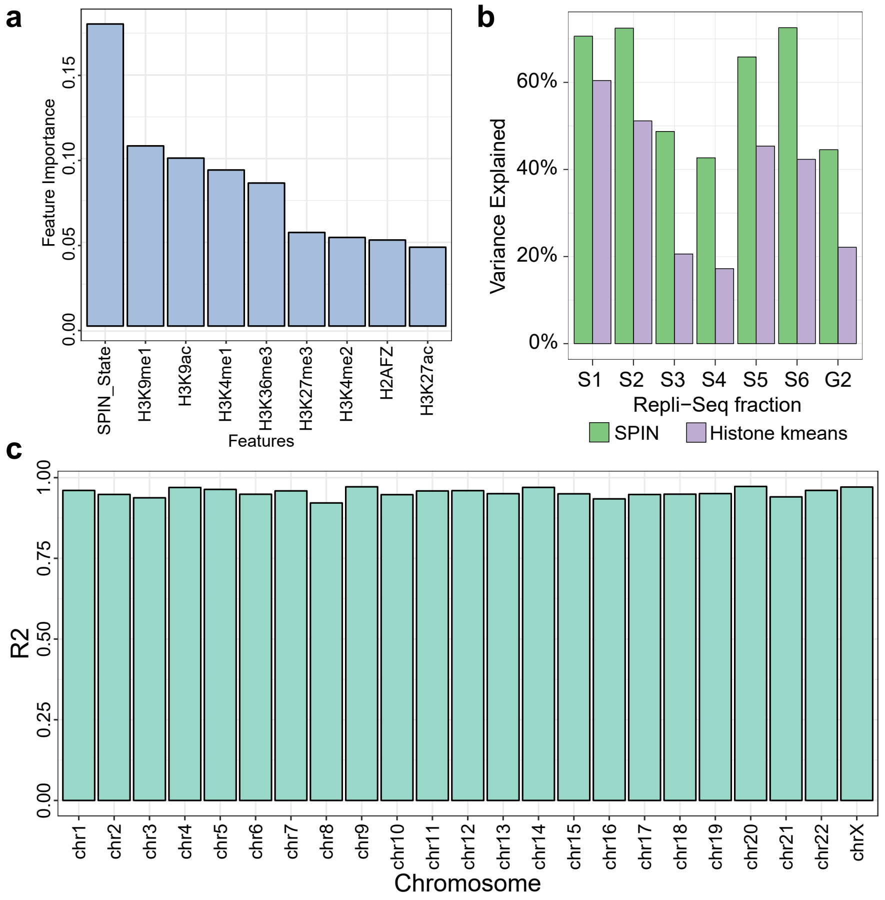

Fig. S6: Evaluation of the model to predict Repli-seq from the SPIN states. **a.** Feature importance of the random forest model to predict Repli-seq signals. **b.** Variance of Repli-seq data explained by the SPIN states and histone marks. Histone marks are categorized using K-means clustering (K=10). **c.** Cross-validation result of predicting Repli-seq. X-axis shows which chromosome is used as test set and the remaining chromosomes are used as training set. R^2 score of prediction on the test set is shown in Y-axis.

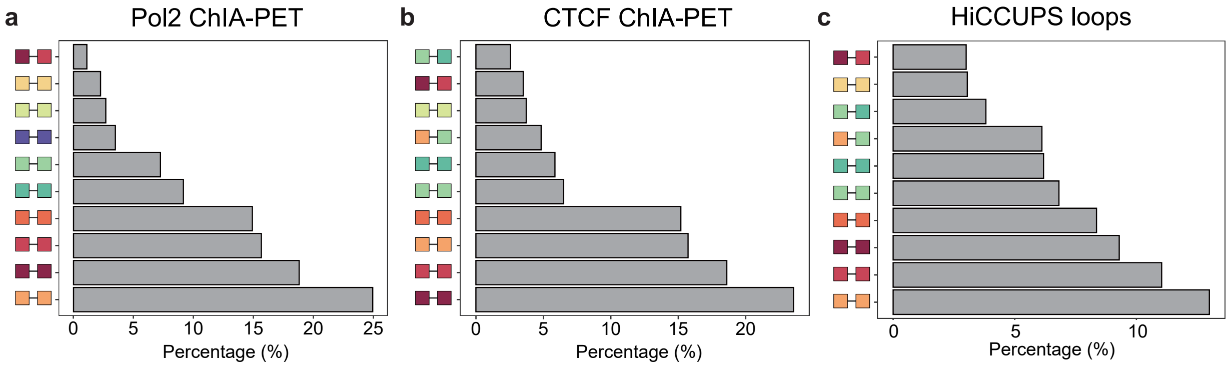

Fig. S7: SPIN states of ChIA-PET significant interaction pairs and HiCCUPS loops. Only ChIA-PET pairs with distance >25kb are considered. X-axis shows the percentage of ChIA-PET or HiCCUPS interactions between 2 specific SPIN states. **a.** Pol2-mediated ChIA-PET loops in K562. **b.** CTCF-mediated ChIA-PET loops in K562. **c.** HiCCUPS loops in K562.

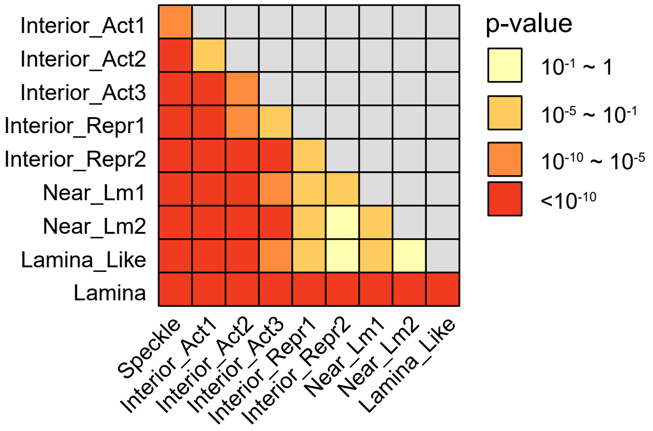

Fig. S8: Wilcoxon Rank-Sum tests on TADs size for different pairs of SPIN states (see Fig. 4c). Bonferroni corrected *p*-values are grouped into 4 intervals and are shown in different colors.

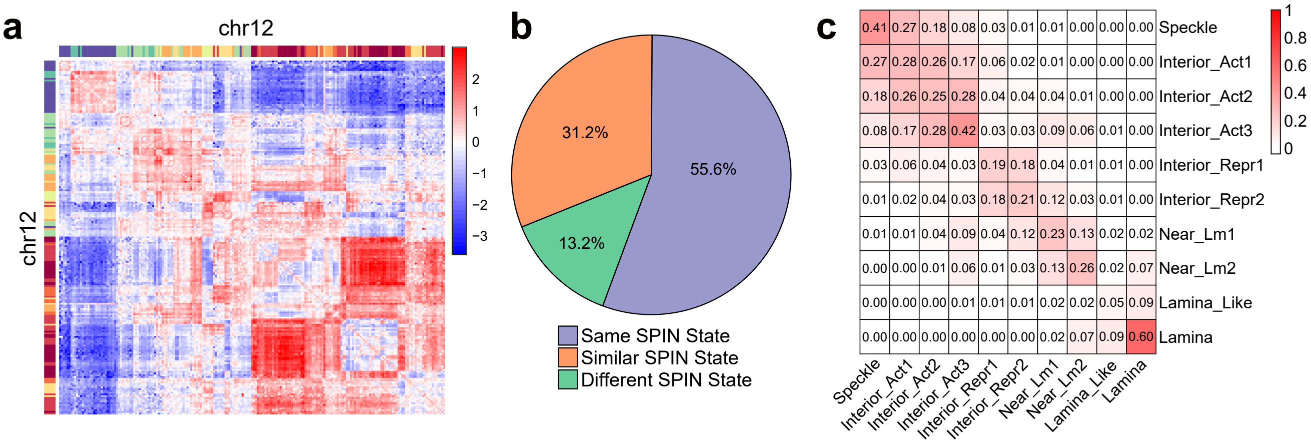

Fig. S9: TAD-TAD interactions among SPIN states. We calculated the median O/E interactions between TADs in K562 (called from the directionality index (DI) method). **a.** Hi-C O/E matrix of TAD-TAD interactions on chr12. **b.** Pie chart showing the percentages of SPIN states among TAD-TAD interactions. **c.** Confusion matrix of TAD-TAD interactions between SPIN states.

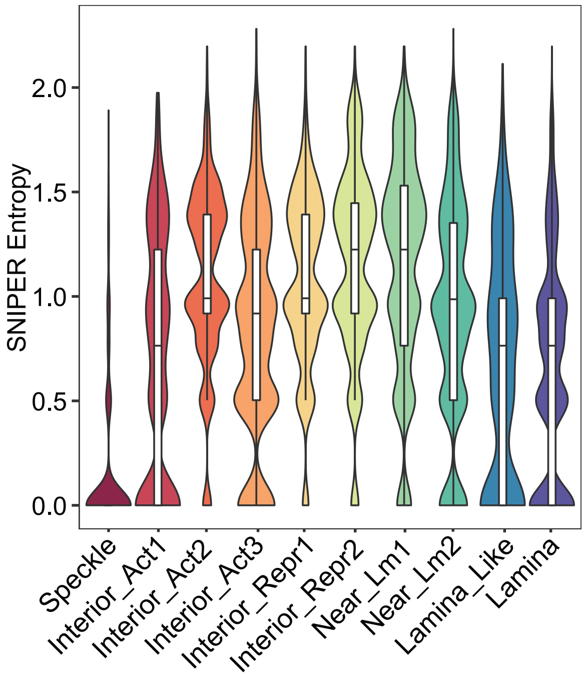

Fig. S10: Violin plot of the entropy for SNIPER Hi-C subcompartments across cell types for different SPIN states (labeled by color). Besides violin plot, the corresponding boxplots are also shown. The entropy is calculated based on SNIPER subcompartment in 9 human cell lines (see Methods).

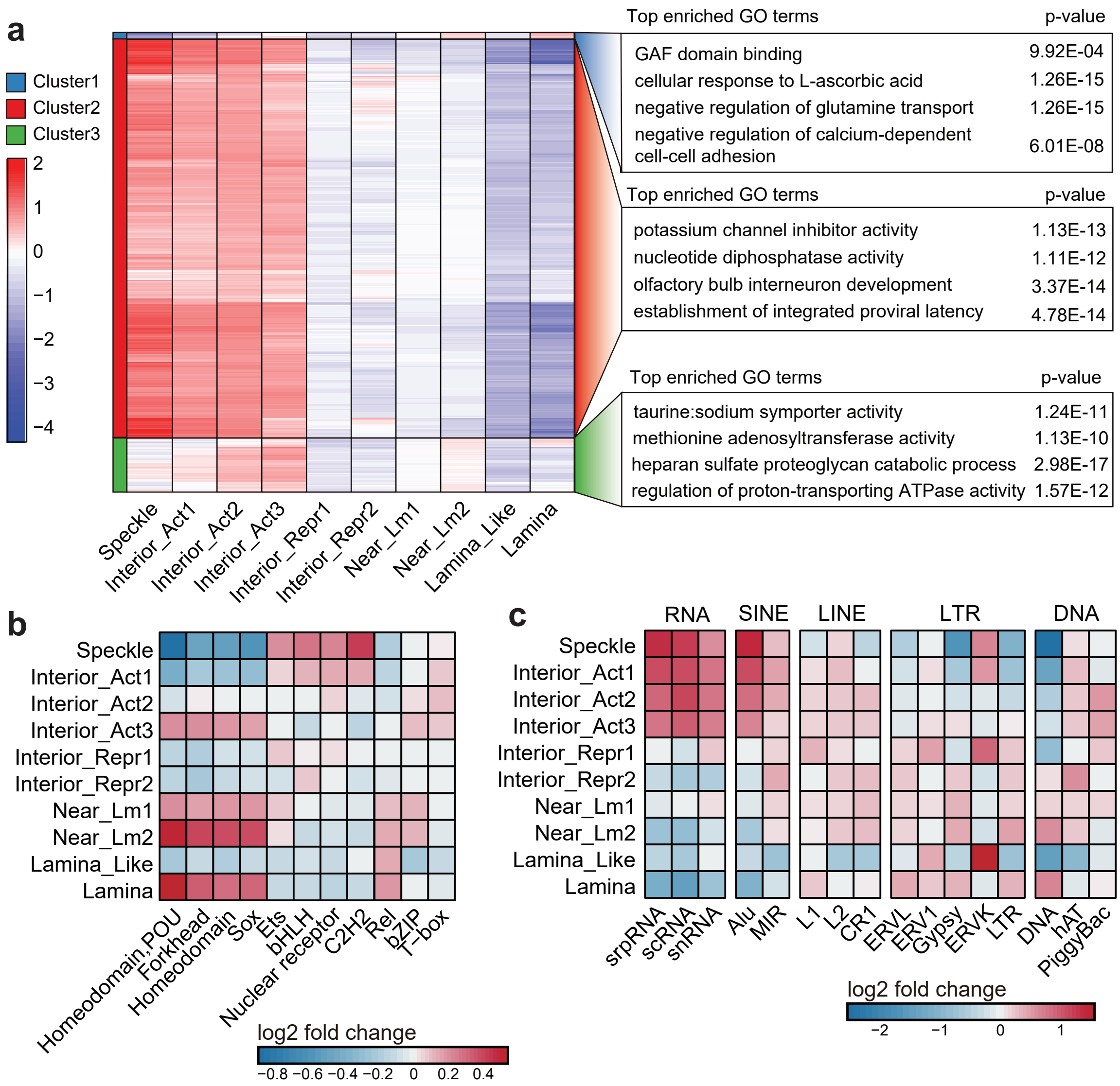

Fig. S11: SPIN states stratify transcriptional regulation and reveal potential sequence features modulating compartmentalization. **a.** Left panel: Heatmap showing ChIP-seq peak enrichment in different SPIN states. 381 ChIP-Seq data sets are used and grouped into 3 clusters. Right panel: GO term enrichment of target genes in each cluster. **b.** Heatmap showing different transcription factor motif families enriched in each SPIN state. Motif families annotations are collected from CIS-BP. log2 fold enrichment of each motif family on SPIN states is shown. **c.** Heatmap showing different repeat families enriched in each SPIN state. Repeat families annotations are collected from the UCSC Genome Browser. log2 fold enrichment of each repeat family on SPIN states is shown.

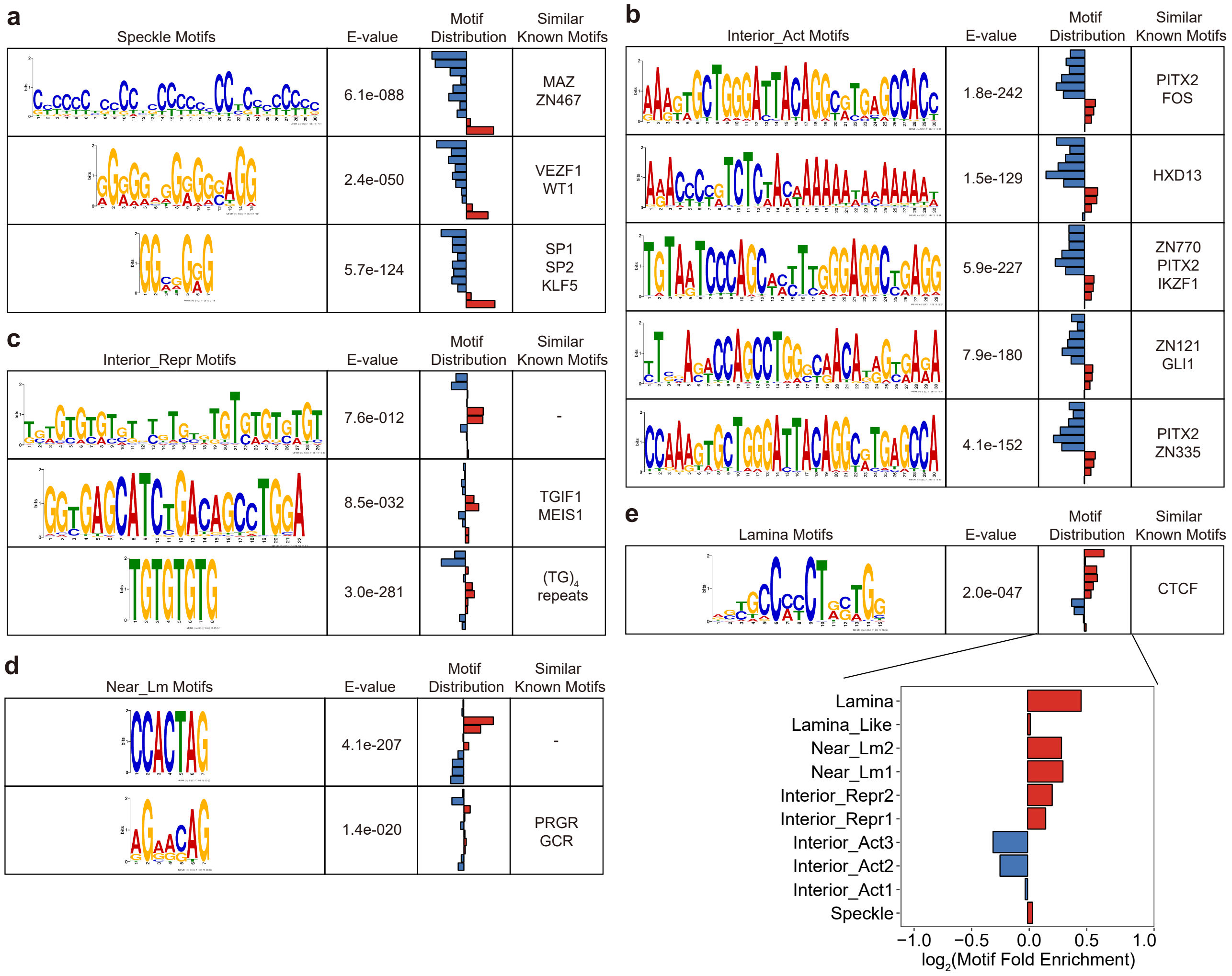

Fig. S12: *De novo* motif discovery on different SPIN states: **a.** Speckle; **b.** Interior_Act; **c.** Interior_Repr; **d.** Near_Lm; and **e.** Lamina. We use MEME to perform motif discovery on each SPIN state. For each motif, the fold enrichment on each SPIN state is shown along with E-values. The *de novo* discovered motifs are matched to known motifs using TomTom.

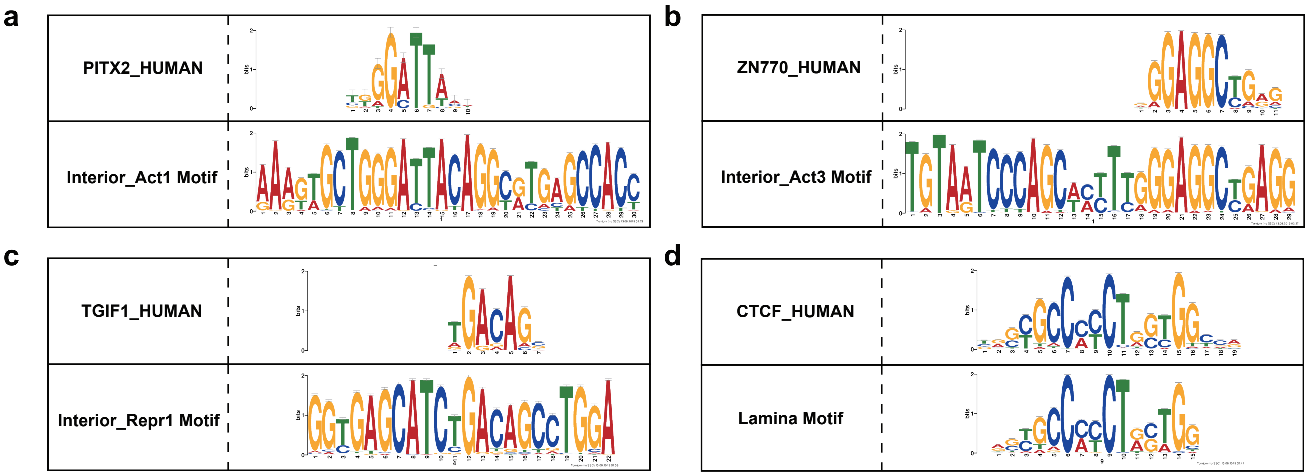

Fig. S13: Match *de novo* discovered motifs to known motifs. Motifs discovered are compared to known motifs from JASPAR by using Tomtom from the MEME Suite. **a.** Motif enriched in the Interior_Act1 state compared with the PITX2 motif. **b.** Motif enriched in the Interior_Act3 state compared with the ZN770 motif. **c.** Motif enriched in the Interior_Repr1 state compared with the TGIF1 motif. **d.** Motif enriched in the Lamina state compared with the CTCF motif.

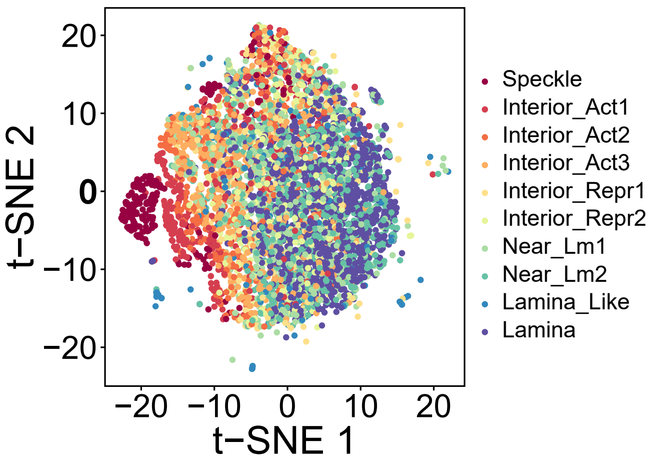

Fig. S14: Visualization of 6-mer occurrences in sequences within different SPIN states. The 6-mer occurrences are normalized by average GC content in each SPIN state. Here we project the normalized 6-mer occurrences onto 2D plane using t-Distributed Stochastic Neighbor Embedding (t-SNE). Each dot is a 25kb bin and the color represents different SPIN states.

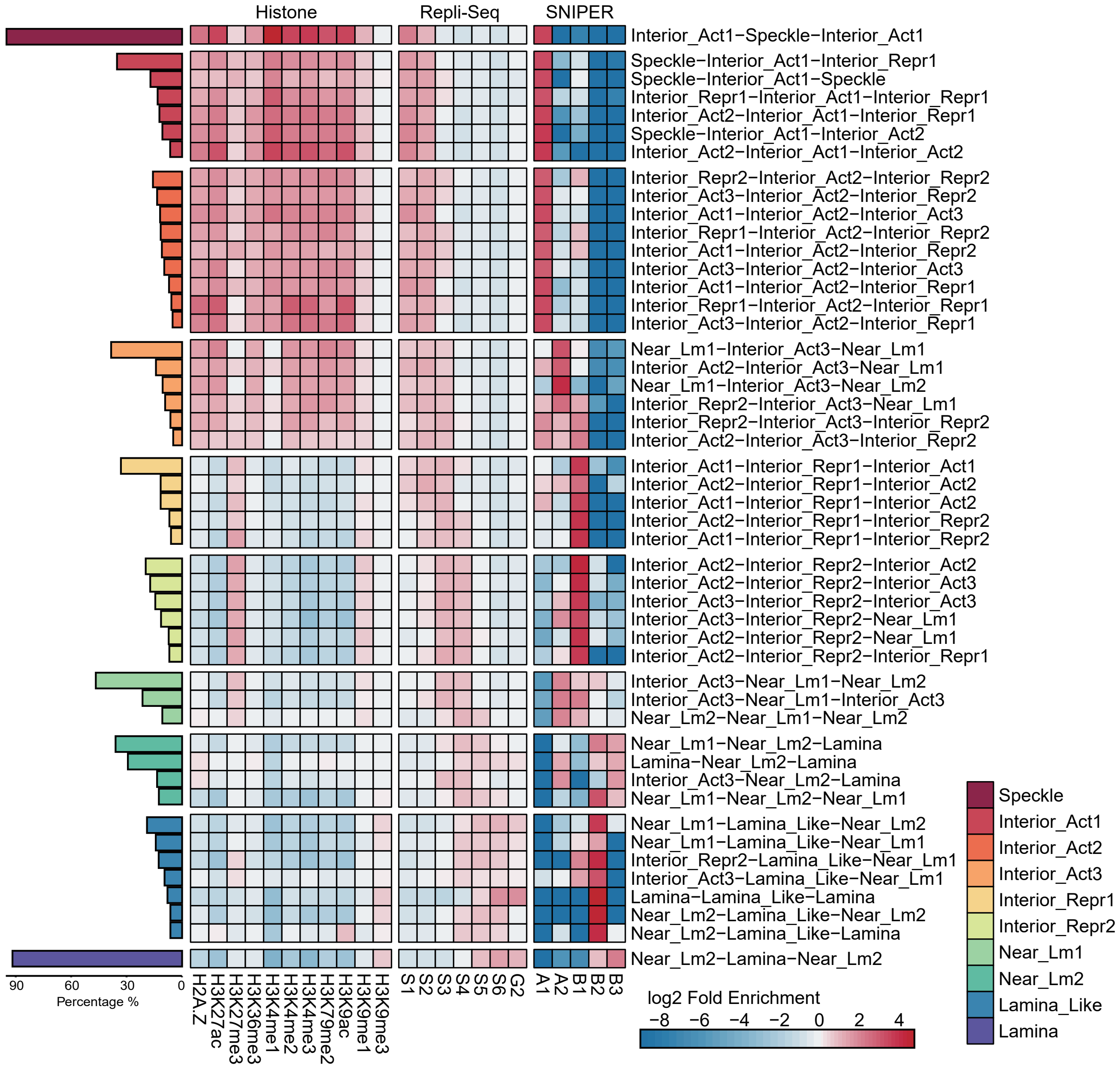

Fig. S15: Heatmap showing the correlation between 3 consecutive SPIN states with histone mark signals, Repli-seq, and Hi-C subcompartments. The rows in the heat map represent different 3 SPIN patterns and are grouped by the SPIN states in the middle. Percentage of each 3 consecutive pattern for each SPIN state is shown on the left. Symmetrical 3 consecutive SPIN patterns are merged, and 3 consecutive SPIN patterns with length <100kb are not include. The heatmap shows the average fold enrichment of genomic signals on the middle SPIN state of each 3 consecutive pattern.

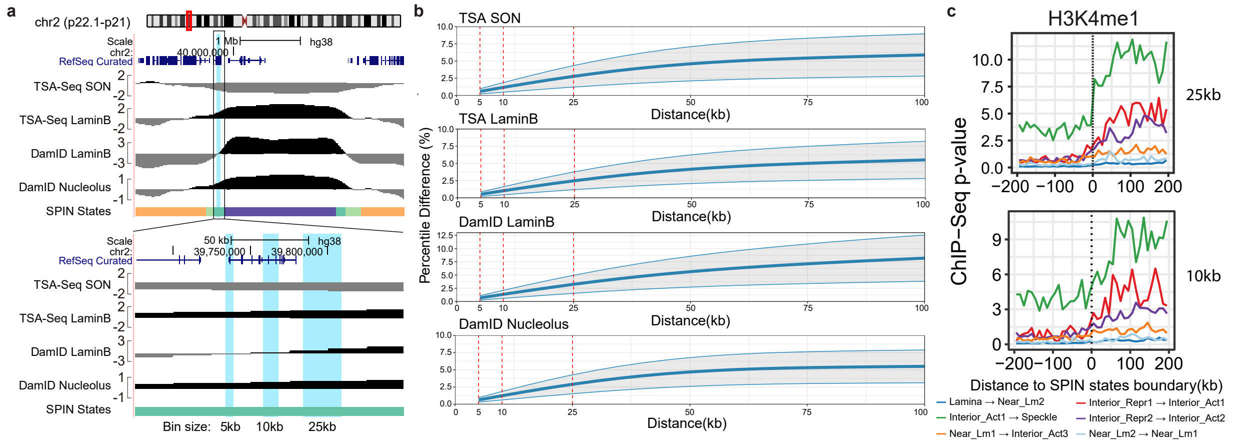

Fig. S16: Justification of the 25kb resolution use in this work. **a.** Genome Browser view of TSA-seq/DamID and SPIN states. In the zoom-in view, three genomic regions with different size (5kb, 10kb, and 25kb) are highlighted. Note that the differences of TSA-seq and DamID scores between neighboring 5kb and 10kb bins are barely noticeable. **b.** The relationship between genomic distance and TSA-seq/DamID scores. X-axis shows the genomic distance of two genomic bin (kb), and Y-axis shows the mean and variance of their TSA-seq/DamID score differences in percentile. 5kb, 10kb, and 25kb are highlighted in red dash lines. **c.** H3K4me1 histone modification signals at the SPIN states boundaries of different resolution. Top panel shows the SPIN states boundaries called at 25kb resolution (used in the main text). Bottom panel shows the SPIN states boundaries called at 10kb resolution.

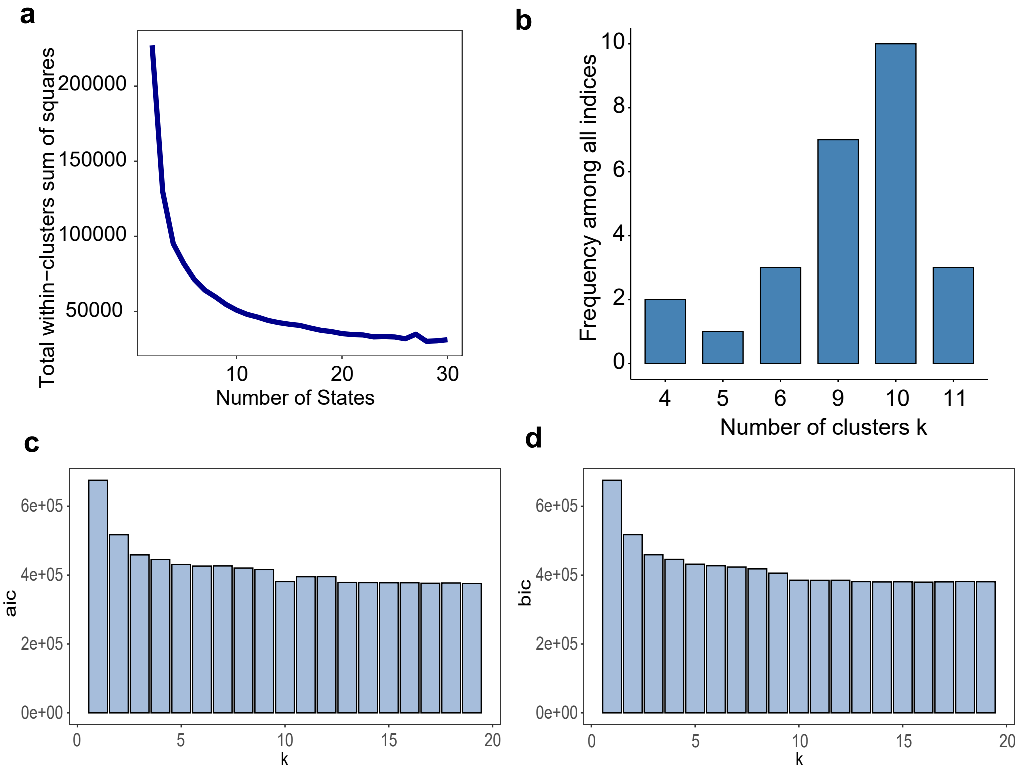

Fig. S17: Justification of the number of states. **a.** To determine the optimal number of states, we first used K-means clustering algorithm to cluster 25kb bins based on TSA-seq and DamID signals. We chose different number of states (k), ranging from 2 to 30. Then we calculated the total within-clusters sum of squares for each clustering results. **b.** Summary of results from NbClust. The histogram shows how many times NbClust proposes each number of states as the best choice. **c.** AIC score of the SPIN results calculated for different number of states. **d.** BIC score of the SPIN results calculated for different number of states.

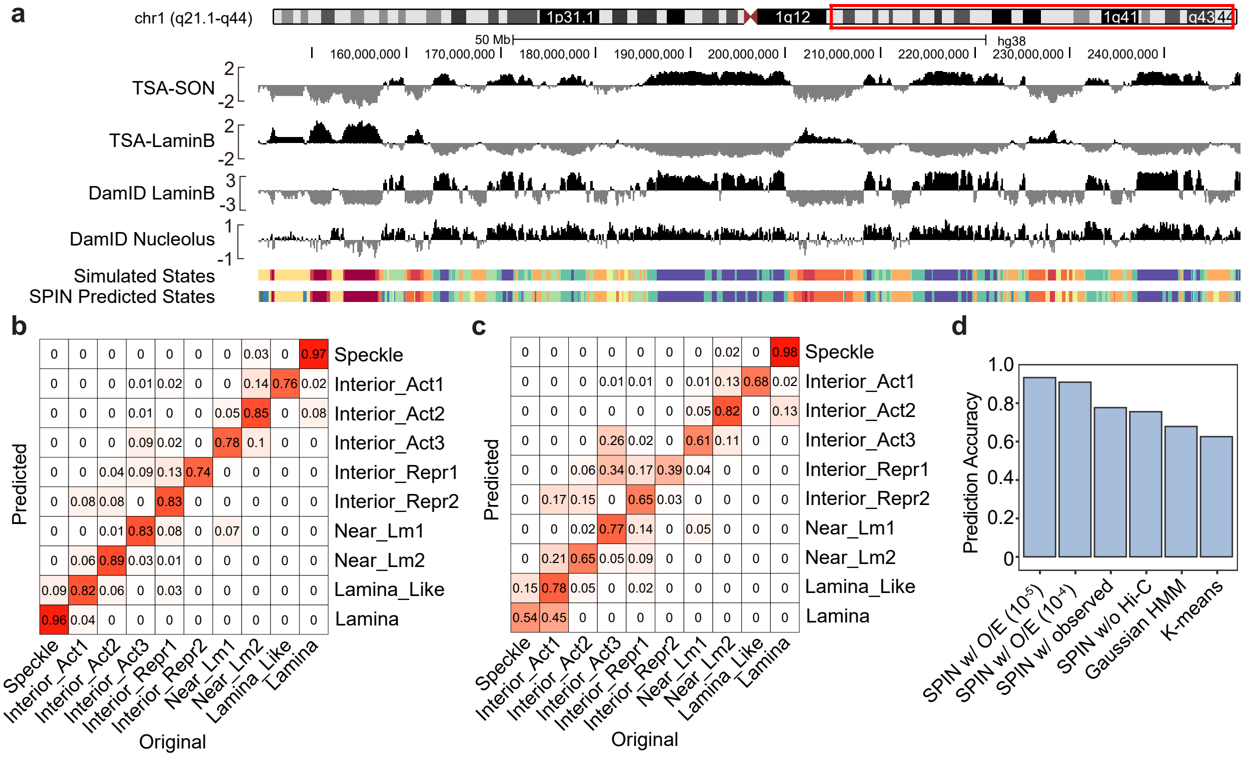

Fig. S18: Results of SPIN on simulated data. **a.** An example of the simulated data. Tracks shown here are (from top to bottom) the simulated SON TSA-seq, Lamin-B1 TSA-seq, Lamin-B1 DamID, Nucleolus DamID signal, the simulated states, and the SPIN predicted states. **b.** Confusion matrix of the SPIN predicted states using Hi-C O/E as edges with *p*-value cutoff 1E-5. The columns represent the true states and the rows show the predicted states. **c.** Confusion matrix of the SPIN predicted states without using Hi-C data as input. **d.** Prediction accuracy of different methods and settings based on the simulated data. The methods used include: SPIN with Hi-C O/E *p*-value cutoff 1E-5, SPIN with Hi-C O/E *p*-value cutoff 1E-4, SPIN with Hi-C observed data, SPIN without using Hi-C data, Gaussian HMM, and K-means.

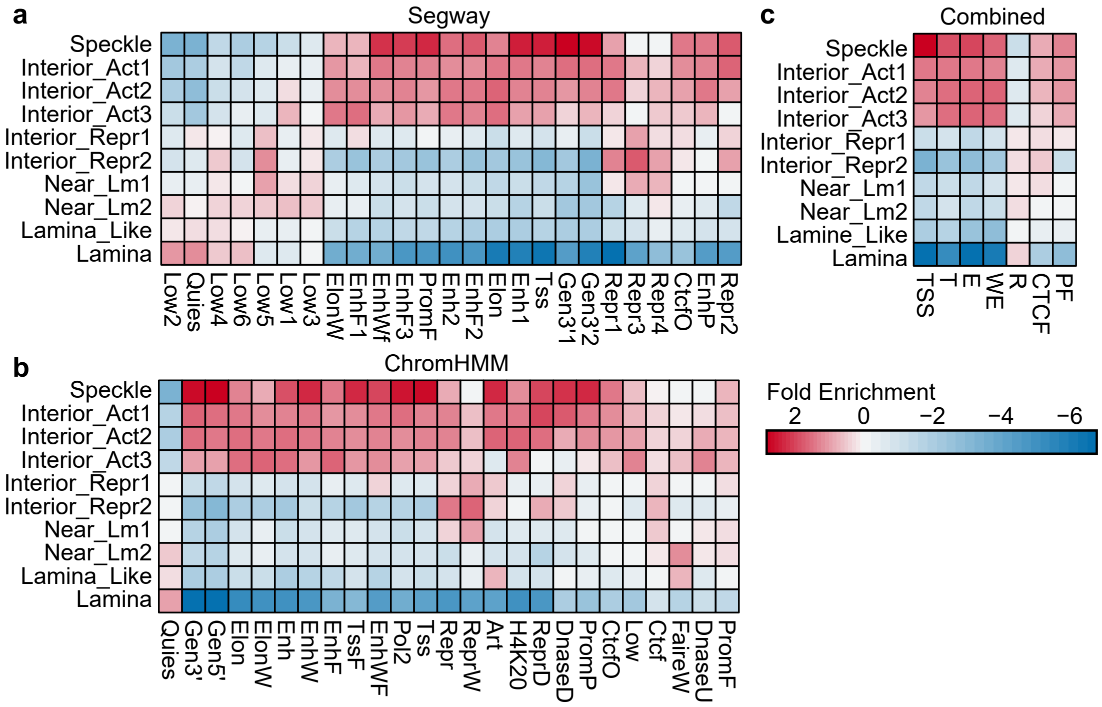

Fig. S19: Comparison with ChromHMM and Segway chromatin states. The heatmap shows the correlation between SPIN states and the chromatin states from ChromHMM, Segway, and the combined segmentation. **a.** Comparison with Segway states in K562. **b.** Comparison with ChromHMM states in K562. **c.** Comparison with combined states from Segway and ChromHMM. For each ChromHMM/Segway/Combined state, fold enrichment score on each SPIN state is calculated.

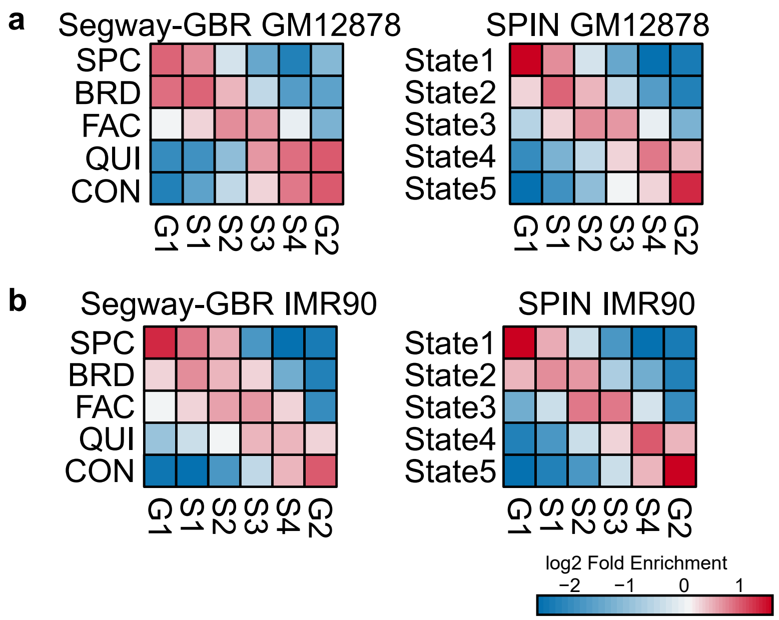

Fig. S20: Comparison between SPIN and Segway-GBR. We used the same input data by Segway-GBR in GM12878 and IMR90 to run SPIN and set the number of states to 5. The heatmaps show the fold enrichment of Repli-seq signals in GM12878 and IMR90 stratified by the SPIN and Segway-GBR states, respectively. **a.** Comparison in GM12878. **b.** Comparison in IMR90.

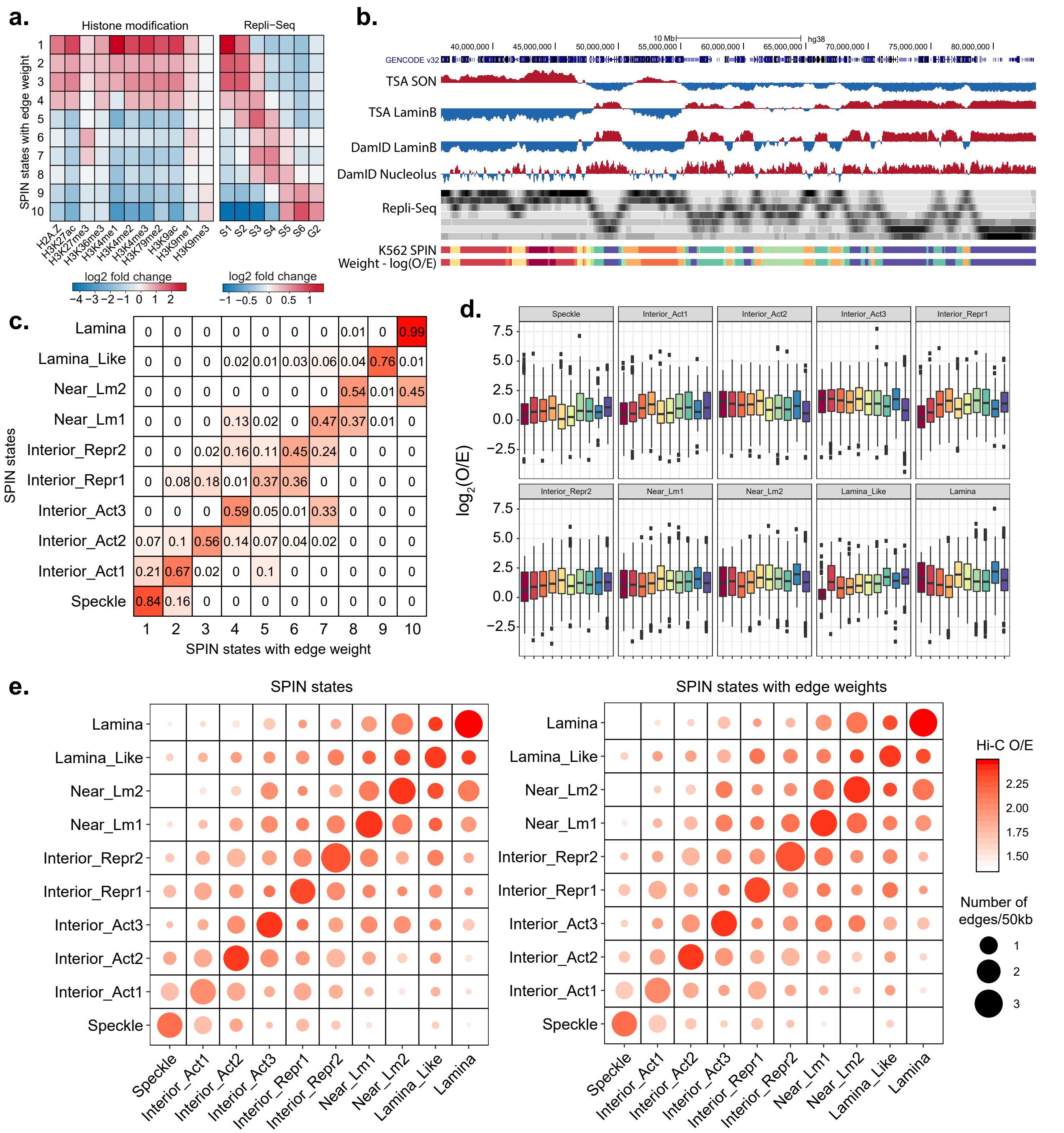

Fig. S21: Incorporating the strengths of Hi-C interactions into the SPIN framework. **a.** Stratification of histone modification and multi-fraction Repli-seq as compared to the SPIN states after adding $\log_2$(O/E) as edge weight. **b.** Genome Browser example of the SPIN states with edge weights added. **c.** Confusion matrix between SPIN states after adding $\log_2$(O/E) as edge weight (X-axis) and the original SPIN states (Y-axis). **d.** Distribution of $\log_2$(O/E) score between SPIN states. **e.** Intra-chromosomal Hi-C interaction O/E strength and the number of edges for each pair of SPIN states. Hi-C interactions with distance $<$100kb are discarded. The average strength of Hi-C O/E is represented by the color intensity of circles and the number of edges is represented by the size of circles. The numbers of edges are normalized by the size of each SPIN states. The heatmap on the left is plotted with the original SPIN states and the one on the right is plotted with SPIN states after adding $\log_2$(O/E) as edge weight.

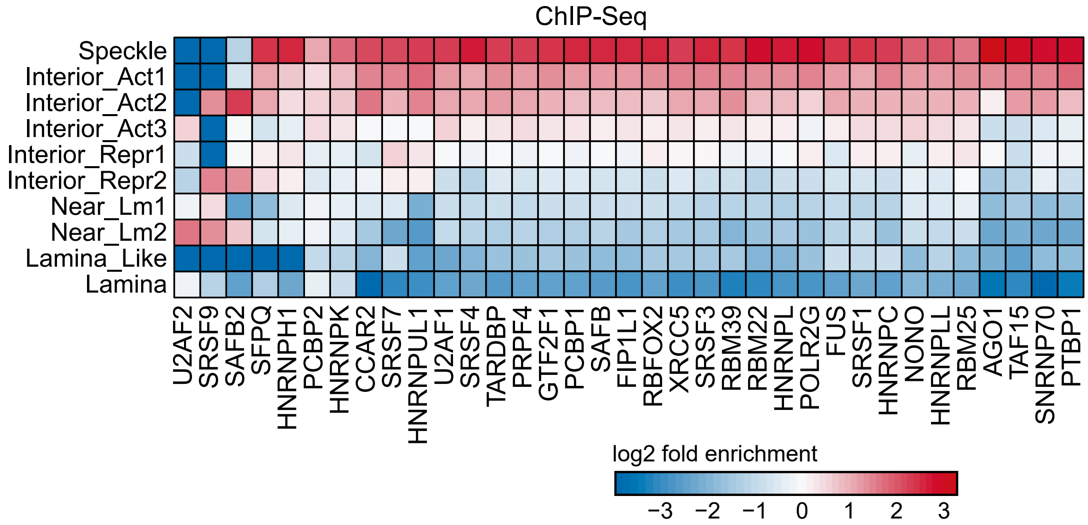

Fig. S22: Heatmap showing RBP binding stratified by different SPIN states. The RBP ChIP-seq datasets were collected from [26]. Log2 fold enrichment ChIP-seq peak density over expected is calculated.
