# Supplementary material for: SPIN reveals genome-wide landscape of nuclear compartmentalization
Source: Genome Biol. 2021 Jan 14;22:36. doi: 10.1186/s13059-020-02253-3 (PMC7809771; doi:10.1186/s13059-020-02253-3)
Supplement: Supplementary file 6 — Additional file 6 Review history. [file 13059_2020_2253_MOESM6_ESM.docx]

Review History

**First round of review**

**Reviewer 1**

**Were you able to assess all statistics in the manuscript, including the appropriateness of statistical tests used?**

Yes, and I have assessed the statistics in my report.

**Comments to author:**

In this paper, the authors developed a new computational method, SPIN, to identify genome-wide nuclear compartmentalization. This work addresses an important research question. SPIN integrates TSA-seq, DamID and Hi-C data to predict 10 states of intranuclear chromosome positioning. The methodology is novel and interesting. The authors performed comprehensive analysis and the data is solid and convincing. The paper is also well written. SPIN has the potential to become a highly useful tool for the research community. I just have a few comments and suggestions which may further improve the current manuscript. Here are my specific comments.

Major comments:

1. As a proof-of-principle, the authors apply SPIN to identify 10 spatial compartmentalization states in K562 cells. It would be very interesting to apply SPIN to other non-cancer cells, such as H1 cells and GM12878 cells, where rich epigenetic datasets and Hi-C datasets are available. Which genomic regions have cell-type-shared and cell-type-specific SPIN states? Whether the cell-type-specific SPIN states are associated with cell-type-specific gene expression? A thorough evaluation of these two questions can further strengthen the biological interpretation of the predicted SPIN states.

2. The authors predicted SPIN state for each 25Kb bin. Is it possible to achieve higher resolution, such as 10Kb or 5Kb bins, which can more precisely characterize the transition of different states? At different bin resolutions, are the SPIN states consistent? In Rao et al 2014 paper, HiCCUPS identified chromatin loops are at 5Kb and 10Kb bin resolutions. Integrating 5Kb and 10Kb resolution Hi-C data into the SPIN model can help to improve the resolution of SPIN states.

3. The authors may incorporate the strength of chromatin interactions into the SPIN model. For example, two bins with stronger Hi-C contact may be more likely to share the same SPIN state. In other word, they can use the significance of Hi-C contact frequency as the weight of each edge.

4. In Figure 4e, the authors showed the SNIPER-predicted subcompartments across 9 cell types. I would expect to see the SPIN states among 9 cell types, which can better catch cell-type-specific chromatin spatial localization.

5. The software GitHub webpage is over-simplified. The authors can add detailed user tutorial and sample input and output files. The authors can also provide more details on the computational cost, including memory and time. How long it takes to infer genome-wide SPIN states?

Minor comment:

1. In Figure S1, if they used different biological replicates of Hi-C data, can they achieve consistent SPIN state?

2. The authors used the EM algorithm to estimate parameters in the model. EM algorithm is deterministic and may be sensitive to initial values for complex model. The authors can perform some sensitivity analysis to evaluate whether the predicted SPIN states are consistent at different initial values. Is it possible that likelihood of the SPIN model consists of multiple local optimal values, and different initial values can lead to different SPIN states with distinct biological interpretation?

3. Page 9, line 43. The authors compared SPIN states with ChIA-PET chromatin loops. In addition to ChIA-PET loops, the authors may also compare with HiCCUPS-identified chromatin loops from Hi-C data, which are more conservative.

4. In Figure 3d, at the bottom of the genome browser view, what is the color scale of 7-fraction Repli-Seq and predicted Repli-Seq? Also in Figure S7a, what is the color scale of Hi-C O/E matrix?

5. Typo: Page 15, line 9. "mean-fied" should be "mean-field".

**Reviewer 2**

**Were you able to assess all statistics in the manuscript, including the appropriateness of statistical tests used?**

No, I do not feel adequately qualified to assess the statistics.

**Comments to author:**

The manuscript "SPIN reveals genome-wide landscape of nuclear compartmentalization" by Wang et al. describes a method, SPIN, to identify spatial compartmentalization states by integrating nuclear compartment mapping data (TSA-seq, in part developed by the senior author, DamID) and chromatin interaction data (Hi-C). SPIN is based on hidden Markov random field, the method utilized by authors in previous publications. It is conceptually similar to (and benchmarked against) ChromHMM, Segway chromatin segmentation tools. Its innovation is the consideration of Hi-C interactions.

Applied to data from K562 cells, the manuscript reports 10 states, associated with distinct features and properties of the genome (Histone marks, gene expression, replication data, TAD/loop boundaries). SPIN segmentation is best in predicting the replication score. Most of the data has been previously published, and one DamID dataset is newly generated.

Supplementary Material reads like an additional paper with more details, observations, tables, and figures. There, data simulation is described, consideration of structural variations, sequence (motif) analysis of SPIN states, details of transitions of chromatin states (trajectories).

\# Major

- Why 10 states? It is described in Methods, but given 10 states is the foundation of the paper, it should be justified in Results.

- Figure S14 is not proof of why the number of states is 10. Why not use more principled methods, like second derivative equal to 0? Subjectively, the inflection point may be ~5, in which case SPIN states may well reflect the known A1, A2, B1-3 compartments

- Methods state: "We found that the appropriate number of states may range from 10 to 15 (Fig. S14). However, results with different state number within the range showed minor difference". Difference in what?

- The number of states issue propagates virtually in any figure.

- Figure 1C - the log2 enrichment scores for some pairs/triplets of states look very similar. Even this figure show distribution box plots, they are not compared with each other. A supplementary heatmap of pairwise comparisons using Wilcoxon and displaying -log10 p-values would be informative in judging which states are significantly different from each other, and which are not.

- Figure 2A - also shows enrichment similarities. Differences also observed, but the n=1 (one cell line) limits what can be said about statistical significance.

- Figure 3A shows that pairs/triples similarity.

- Figure 4C, like figure 1C, don't have pairwise statistical comparisons.

- The abstract reads like it presents a new method, SPIN. SPIN is presented and well-described; however, the paper's primary focus is on the characterization of states SPIN is detecting. Given the nature of data SPIN utilizes, it seems to have a very narrow range of data-dependent applications, not a general method. Shifting the focus from method to states would help.

- As a software, the code should be polished. Currently, three Python files are provided. The code is very sparsely commented, making it hard to understand. Some pieces are commented out, raising questions about why they are still present. The description/documentation of the method on GitHub (currently, near absent) should be provided.

\# Minor

- SON TSA-seq signal is mentioned on p.5, l.7., but defined only in the Figure 1 legend

- Figure 2 legend - typo "enrichmenh"

- Figure 2B - would it be better to express transition pattern counts in %? Same with 4B?

- Figure 2C - are these plots aggregate genome-wide information?

**Authors Response**

**Point-by-point responses to the reviewers’ comments:**

In this paper, the authors developed a new computational method, SPIN, to identify genome-wide nuclear compartmentalization. This work addresses an important research question. SPIN integrates TSA-seq, DamID and Hi-C data to predict 10 states of intranuclear chromosome positioning. The methodology is novel and interesting. The authors performed comprehensive analysis and the data is solid and convincing. The paper is also well written. SPIN has the potential to become a highly useful tool for the research community. I just have a few comments and suggestions which may further improve the current manuscript. Here are my specific comments.

Major comments:

1. As a proof-of-principle, the authors apply SPIN to identify 10 spatial compartmentalization states in K562 cells. It would be very interesting to apply SPIN to other non-cancer cells, such as H1 cells and GM12878 cells, where rich epigenetic datasets and Hi-C datasets are available. Which genomic regions have cell-type-shared and cell-type-specific SPIN states? Whether the cell-type-specific SPIN states are associated with cell-type-specific gene expression? A thorough evaluation of these two questions can further strengthen the biological interpretation of the predicted SPIN states.

*Response: We understand this concern from the reviewer. We would like to clarify that the in¬put data for SPIN needs TSA-seq and DamID for multiple nuclear bodies. We have the most data of TSA-seq and DamID in K562 to demonstrate the real data application of the SPIN method in this work. As a Method paper, our detailed analysis of SPIN states by comparing to or-thogonal epigenomic data in K562 and also the comparisons with Hi-C subcompartment annota-tions across multiple cell types provide proof-of- principle demonstration of the unique value and effectiveness of our method.*

*To clarify the reviewer’s concern and demonstrate the general applicability of our method, here we provide preliminary results by applying SPIN to HCT116 cells. Fig. R1 shows the comparison between the SPIN states in HCT116 and epigenomic and replication timing data in HCT116. Similar to what we show in Fig. 2a and Fig. 3a for K562 cells, the SPIN states in HCT116 nicely stratify various histone marks (both active and repressive) and multi-fraction Repli-seq data. This demonstrates the robust-ness and generalizability of our method in other cell types. We are currently indeed actively working on producing SPIN states in other cell types such as HCT116. But conceptually, it needs much more work to tackle a major challenge of comparing SPIN states across cell types in the most biologically meaningful way. More specifically, this is due to the complication caused by different nuclear geome-tries of different cell types, which is a unique problem that no previous cross cell-type genomic data comparison work would need to handle. Therefore, a new subsequent algorithm is needed to optimally derive conserved and cell-type specific nuclear compartmentalization states and their variations across multiple cell types. We are understandably very much reluctant to include SPIN states in other cell types in the current manuscript for comparisons since there is much development needed before we can get this all sorted out to the point we are comfortable to publish the cross cell-type comparisons. We have also communicated our thoughts with the editor.*

*We do have a plan to comprehensively analyze nuclear compartmentalization across many cell types using SPIN in the near future. That will require much more time and effort to collect TSA-seq and DamID data for multiple nuclear bodies in different cell types and, more importantly, to develop additional approaches that can solve for a compendium of SPIN states covering all possible nuclear compartments – including some that are present in all cell types and others that are only present in some cell types – and properly analyze them. Therefore, the request to apply SPIN to more cell types (the later comment #4 on the application to 9 cell types) is currently beyond the scope of this Method paper. We therefore focus on the robustness and effectiveness of our method in this manuscript and will have the cross cell-type SPIN states comparison as a separate manuscript in the near future. As we and others produce more TSA-seq and DamID data, our generalizable method SPIN introduced in this manuscript will greatly facilitate integrative analysis together with Hi-C and provide new interpretation of various other types of genome structural and functional mapping data.*

2. The authors predicted SPIN state for each 25Kb bin. Is it possible to achieve higher resolution, such as 10Kb or 5Kb bins, which can more precisely characterize the transition of different states? At different bin resolutions, are the SPIN states consistent? In Rao et al 2014 paper, HiCCUPS identified chromatin loops are at 5Kb and 10Kb bin resolutions. Integrating 5Kb and 10Kb resolution Hi-C data into the SPIN model can help to improve the resolution of SPIN states.

*Response: This is an important suggestion. We would like to clarify that the Hi-C data we used in this paper were processed with Juicer tools in predetermined resolutions that are readily available (5kb, 10kb, 25kb, 50kb, etc.). Our SPIN framework can in principle be applied to higher resolution than 25kb, such as 5kb and 10kb. However, the goal of SPIN is to provide a genome-wide identification of nuclear compartmentalization patterns which have very different resolution than local chromatin state (e.g., ChromHMM) and loop anchors (from HiCCUPS). Crucially, our input data types (TSA-seq and DamID) are in resolutions much lower than 10kb. For example, the TSA-seq scores are in 20kb resolution. In addition, as an example shown in Fig. S16a-b, at 5kb and 10kb resolutions, the differences of TSA-seq and DamID scores between the neighboring bins are too small to be observed (Δpercentile <1%). Also, higher resolution does not show sharper histone mark transition at SPIN states boundary (Fig. S16c). Together, we found that 25kb resolution is an appropriate resolution for identifying SPIN states in this work. We have also added these analysis and discussions to Supplementary Results with new Fig. S16.*

3. The authors may incorporate the strength of chromatin interactions into the SPIN model. For example, two bins with stronger Hi-C contact may be more likely to share the same SPIN state. In other word, they can use the significance of Hi-C contact frequency as the weight of each edge.

*Response: We thank the reviewer for the suggestion. We further assessed the impact of incorpo-rating interaction strength into the SPIN framework (Supplementary Results and Fig. S21). We found that incorporating Hi-C interaction strength into the SPIN framework does not provide improvement to the identification of compartmentalization patterns (Fig. S21a-c and Supplementary Results). As we discussed in the manuscript, high Hi-C O/E interaction does not necessarily indicate the same state, and the interaction could come from SPIN states with similar, but different, spatial localization context (Fig. S21d-e and Supplementary Results), although there is a general trend that genomic bins with higher Hi-C O/E scores would be more likely to share SPIN states. Our results suggest that the spatial localization state (SPIN state) for a genomic bin is more reflected by its spatial localization neighbors rather than their interaction edge weights. However, to leave this as a possible option to the users, this is now implemented in our source code of SPIN and users can add customized edge weights to the framework if needed for their specific applications and biological questions.*

4. In Figure 4e, the authors showed the SNIPER-predicted subcompartments across 9 cell types. I would expect to see the SPIN states among 9 cell types, which can better catch cell-type-specific chromatin spatial localization.

*Response: We certainly agree that this is an important direction. In fact, it will guide our future effort as our next steps. However, as we explained in our detailed response to the review comment #1 (see above), such cross cell-type SPIN state comparison among 9 cell types is currently not feasible from a logistic standpoint, as that will require much more time and resource to generate TSA-seq and DamID data, and also not yet possible from a technical perspective due to the need for new cross cell-type comparison algorithm to consider nuclear geometries. This would be beyond the scope of this Method paper. In this work, as a proof-of-concept we compared the SPIN states in K562 with the SNIPER-predicted Hi-C subcompartments (which also indirectly correlate with spatial position patterns). Even though this is not a direct comparison and cannot provide cell-type specific SPIN states, this analysis nevertheless has derived novel insights into the general conservation of spatial compartmentalization patterns and paved the way for future cross cell-type SPIN states comparative analysis when the data and approaches are available.*

5. The software GitHub webpage is over-simplified. The authors can add detailed user tutorial and sample input and output files. The authors can also provide more details on the computational cost, including memory and time. How long it takes to infer genome-wide SPIN states?

*Response: We have now significantly improved the documentation of the code on GitHub on how to run the code with a detailed example. The SPIN framework was tested on a Linux server with Intel(R) Xeon(R) CPU E5-4650 v4 @ 2.20GHz. Without acceleration from multiprocessing parallelism on the algorithm, genome-wide inference of SPIN states took about 6 hours of run time and 13Gb of memory.*

Minor comment:

1. In Figure S1, if they used different biological replicates of Hi-C data, can they achieve consistent SPIN state?

*Response: We have added the results of using two different Hi-C replicates in Fig. S1a. The two replicates of Hi-C data in K562 were downloaded from the 4DN data portal. We kept other input data the same except for different Hi-C datasets. As shown in Fig. S1a, when using different Hi-C replicates as input, we can still achieve consistent SPIN states (accuracy >94%).*

2. The authors used the EM algorithm to estimate parameters in the model. EM algorithm is deterministic and may be sensitive to initial values for complex model. The authors can perform some sensitivity analysis to evaluate whether the predicted SPIN states are consistent at different initial values. Is it possible that likelihood of the SPIN model consists of multiple local optimal values, and different initial values can lead to different SPIN states with distinct biological interpretation?

*Response: Following the reviewer’s suggestion, we performed sensitivity analysis to test the robustness of SPIN states. To do that, we randomly initialized the model and assessed whether we can obtain consistent SPIN results. This was done by passing a random seed to the method (K-means) at the step of initializing the parameters, where the initial centroids of K-means was randomly chosen. We calculated micro/macro-F1 scores to measure the consistency of results. As shown in Fig. S1b, we performed 100 randomly initialized experiments, the average of micro/macro-F1 score is above 0.85. This demonstrated that SPIN can achieve consistent results from randomly initialized states, suggesting robustness of the method.*

3. Page 9, line 43. The authors compared SPIN states with ChIA-PET chromatin loops. In addition to ChIA-PET loops, the authors may also compare with HiCCUPS-identified chromatin loops from Hi-C data, which are more conservative.

*Response: In addition to ChIA-PET loops, we have now added the comparison between SPIN states and HiCCUPS Hi-C loops (Fig. S7c). We found that the majority (81.4%) of HiCCUPS loops are formed by genomic loci with the same SPIN states. In addition, there are more HiCCUPS loops detected between the SPIN states more toward the nuclear interior with higher transcriptional activity. These observations are in general highly consistent with the results from ChIA-PET loops (Fig. S7a-b), further confirming that chromatin loops tend to be formed by the genomic loci from the same SPIN states.*

4. In Figure 3d, at the bottom of the genome browser view, what is the color scale of 7-fraction Repli-Seq and predicted Repli-Seq? Also in Figure S7a, what is the color scale of Hi-C O/E matrix?

*Response: We apologize for the confusion. The color scale of 7-fraction Repli-seq is for the normalized Repli-seq signal of each fraction over all fractions. The color scale of Hi-C O/E matrix is the log2O/E Hi-C score. We added the color scale in Fig. 3d and Fig. S9 (original Fig. S7) and clarified in the figure captions.*

5. Typo: Page 15, line 9. "mean-fied" should be "mean-field".

*Response: Fixed.*

**Reviewer 2**

The manuscript "SPIN reveals genome-wide landscape of nuclear compartmentalization" by Wang et al. describes a method, SPIN, to identify spatial compartmentalization states by integrating nuclear compartment mapping data (TSA-seq, in part developed by the senior author, DamID) and chromatin interaction data (Hi-C). SPIN is based on hidden Markov random field, the method utilized by authors in previous publications. It is conceptually similar to (and benchmarked against) ChromHMM, Segway chromatin segmentation tools. Its innovation is the consideration of Hi-C interactions.

Applied to data from K562 cells, the manuscript reports 10 states, associated with distinct features and properties of the genome (Histone marks, gene expression, replication data, TAD/loop boundaries). SPIN segmentation is best in predicting the replication score. Most of the data has been previously published, and one DamID dataset is newly generated.

Supplementary Material reads like an additional paper with more details, observations, tables, and figures. There, data simulation is described, consideration of structural variations, sequence (motif) analysis of SPIN states, details of transitions of chromatin states (trajectories).

\# Major

- Why 10 states? It is described in Methods, but given 10 states is the foundation of the paper, it should be justified in Results.

- Figure S14 is not proof of why the number of states is 10. Why not use more principled methods, like second derivative equal to 0? Subjectively, the inflection point may be ~5, in which case SPIN states may well reflect the known A1, A2, B1-3 compartments

- Methods state: "We found that the appropriate number of states may range from 10 to 15 (Fig. S14). However, results with different state number within the range showed minor difference". Difference in what?

*Response: We appreciate this important suggestion. We agree that choosing the right number of states is crucial. To justify the use of 10 states, we have now added additional analysis based on two more principled strategies: (1) We use Akaike information criterion (AIC) and Bayesian information criterion (BIC) score to determine the appropriate number of states. (2) We use the ‘NbClust’ software package to estimate the best clustering scheme. As shown in Fig. S17 and discussed in the Methods section, both strategies consistently suggest 10 as the number of states. Regarding our original statement of the differences of state numbers ranging from 10 to 15, we were referring to the minor differences in sum of squares of TSA-seq/DamID signals, as shown in the K-means elbow methods (now Fig. S17a). With these additional analysis (Methods section and Fig. S17), we now further demonstrate why 10 is the appropriate number of states for K562 cells in this work.*

*We would like to clarify that the 5 state Hi-C subcompartments were identified from inter-chromosomal Hi-C contact map in GM12878 without using any TSA-Seq/DamID as input. In this work, we show that 5 states Hi-C subcompartment can be greatly refined by SPIN states with spatial interpretation rel¬ative to multiple nuclear bodies. For example, in Fig. 2a, Speckle and Interior Act1 states are most strongly associated with A1 subcompartment even though they have significantly different distribution of TSA-seq/DamID (Fig. S2). The difference is also supported by other genomic features such as hi-stone modification (Fig. 2c and Fig. S5), further suggesting the advantage of SPIN states over Hi-C subcompartments.*

- The number of states issue propagates virtually in any figure.

- Figure 1C - the log2 enrichment scores for some pairs/triplets of states look very similar. Even this figure show distribution box plots, they are not compared with each other. A supplementary heatmap of pairwise comparisons using Wilcoxon and displaying -log10 p-values would be informative in judging which states are significantly different from each other, and which are not.

*Response: We thank the reviewer for this suggestion. As the reviewer suggested, to show that there are significant differences in the distribution of TSA-seq/DamID signals, we performed Wilcoxon tests for each pairs of SPIN states. As shown in Fig. S2, the majority pairs of SPIN states exhibit significant differences (p-value < 10−10) in terms of TSA-seq/DamID signal distributions. Indeed, Interior Repr1 and Interior Act2 have more similar SON TSA-seq distributions (p-value = 0.62) and Interior Repr1 and Interior Repr2 have more similar LaminB TSA-seq and LaminB DamID distributions (10−5 < p-value < 10−1). However, it is important to point out that they have significantly different distributions with other TSA-seq/DamID signals (p-value < 10−10), demonstrating the value of the integrative approach of SPIN and reflecting their different spatial localization patterns in the nucleus.*

- Figure 2A - also shows enrichment similarities. Differences also observed, but the n=1 (one cell line) limits what can be said about statistical significance.

*Response: We have added statistical significance of the difference between neighboring states in Fig. 2a based on Wilcoxon Rank-Sum tests.*

- Figure 3A shows that pairs/triples similarity.

*Response: We have now added statistical significance for neighboring states in Fig. 3a based on Wilcoxon Rank-Sum tests.*

- Figure 4C, like figure 1C, don't have pairwise statistical comparisons.

*Response: For Fig. 4c, we have added Wilcoxon Rank-Sum tests results of TADs size comparison for each pair of SPIN states in Fig. S8.*

- The abstract reads like it presents a new method, SPIN. SPIN is presented and well-described; however, the paper's primary focus is on the characterization of states SPIN is detecting. Given the nature of data SPIN utilizes, it seems to have a very narrow range of data-dependent applications, not a general method. Shifting the focus from method to states would help.

*Response: We appreciate the reviewer’s comment. As mentioned in the editor’s decision letter, our manuscript best fits the Method category of the article. We have thus revised the manuscript accordingly. Regarding the nature of the SPIN method, it is important to note our method is a generalizable new approach for analyzing genome-wide nuclear compartmentalization. The application to K562 in this manuscript serves as a proof-of-principle demonstration of the SPIN method, mostly because we have the most data of TSA-seq and DamID in K562 at this point. Our detailed analysis of SPIN states by comparing to abundant, orthogonal epigenomic data in K562 further demonstrate the unique value and effectiveness of our method. As we explained in our response to review comment #1 from Reviewer #1, we have a plan to comprehensively analyze nuclear compartmentalization across multiple cell types using SPIN in the near future.*

- As a software, the code should be polished. Currently, three Python files are provided. The code is very sparsely commented, making it hard to understand. Some pieces are commented out, raising questions about why they are still present. The description/documentation of the method on GitHub (currently, near absent) should be provided.

*Response: We appreciate this important comment from the reviewer. We have made effort to sig-nificantly improve the accessibility of the code and also the documentation: (1) We have polished the comments in the source code to improve readability. (2) We have greatly improved the README docu¬mentation to explain how to run the code with a detailed example.*

\# Minor

- SON TSA-seq signal is mentioned on p.5, l.7., but defined only in the Figure 1 legend

*Response: We apologize for the confusion. We have added text in the main text when we first introduce the input data to clarify that the SON protein was used by TSA-seq to map genome-wide distance to nuclear speckles.*

- Figure 2 legend - typo "enrichmenh"

*Response: Fixed.*

- Figure 2B - would it be better to express transition pattern counts in %? Same with 4B?

*Response: We appreciate this suggestion. We have now changed the count into percentage in Fig. 2b, Fig. 4b, and Fig. S7.*

- Figure 2C - are these plots aggregate genome-wide information?

*Response: Yes, they are aggregated from genome-wide signal changes across state boundaries.*

**Second round of review**

**Reviewer 1**

The authors did a great job in the revision and fully addressed my previous comments and suggestions. The paper has been significantly improved. I don't have further comments and think the current version is ready for publication.
